# *Sulfobacillus thermotolerans*: new insights into resistance and metabolic capacities of acidophilic chemolithotrophs

**DOI:** 10.1038/s41598-019-51486-1

**Published:** 2019-10-21

**Authors:** Anna E. Panyushkina, Vladislav V. Babenko, Anastasia S. Nikitina, Oksana V. Selezneva, Iraida A. Tsaplina, Maria A. Letarova, Elena S. Kostryukova, Andrey V. Letarov

**Affiliations:** 1 Research Center of Biotechnology of the Russian Academy of Sciences, Winogradsky Institute of Microbiology, Moscow, 119071 Russia; 2 Federal Medical Biological Agency, Federal Research and Clinical Center of Physical-Chemical Medicine, Moscow, 119435 Russia

**Keywords:** Applied microbiology, Bacterial genomics

## Abstract

The first complete genome of the biotechnologically important species *Sulfobacillus thermotolerans* has been sequenced. Its 3 317 203-bp chromosome contains an 83 269-bp plasmid-like region, which carries heavy metal resistance determinants and the rusticyanin gene. Plasmid-mediated metal resistance is unusual for acidophilic chemolithotrophs. Moreover, most of their plasmids are cryptic and do not contribute to the phenotype of the host cells. A polyphosphate-based mechanism of metal resistance, which has been previously unknown in the genus *Sulfobacillus* or other Gram-positive chemolithotrophs, potentially operates in two *Sulfobacillus* species. The methylcitrate cycle typical for pathogens and identified in the genus *Sulfobacillus* for the first time can fulfill the energy and/or protective function in *S. thermotolerans* Kr1 and two other *Sulfobacillus* species, which have incomplete glyoxylate cycles. It is notable that the TCA cycle, disrupted in all *Sulfobacillus* isolates under optimal growth conditions, proved to be complete in the cells enduring temperature stress. An efficient antioxidant defense system gives *S. thermotolerans* another competitive advantage in the microbial communities inhabiting acidic metal-rich environments. The genomic comparisons revealed 80 unique genes in the strain Kr1, including those involved in lactose/galactose catabolism. The results provide new insights into metabolism and resistance mechanisms in the *Sulfobacillus* genus and other acidophiles.

## Introduction

Extremely acidophilic chemolithotrophic microorganisms (ACM) form a unique group of polyextremophiles due to their ability to adapt to diverse extreme factors, including exceptionally low pH (≤1) and high concentrations of toxic heavy metals and metalloids. These microorganisms are present in ore deposits, coal mines, acid hot springs, hydrothermal vents, and acid mine drainage^1^. Ferrous iron (Fe^2+^), elemental sulfur (S^0^), reduced sulfur compounds (S^2−^), and hydrogen serve as inorganic electron donors and energy sources in different ACM. These characteristics make it possible to use the communities of acidophilic chemolithotrophic bacteria and archaea in biohydrometallurgical (biomining) approaches for the recovery of precious and non-ferrous metals^2–6^. Nowadays, biomining is applied worldwide and recognized as a more environmentally friendly and economically advantageous method for processing of sulfide raw materials.

Bacteria of the genus *Sulfobacillus*, which are common inhabitants of extremely acidic metal-rich ecological niches, are successfully used in the bioleaching/biooxidation of sulfide ores (including refractory pyrite–arsenopyrite gold-bearing ores) and ore concentrates. Members of the genus *Sulfobacillus* play a leading role in both Fe^2+^ and S^0^/S^2−^ oxidation in the communities of ACM^1,7,8^. Previous studies of carbon and energy metabolism have shown that bacteria of the genus *Sulfobacillus* are facultative chemolithotrophs with an optimal mixotrophic type of growth in the presence of organic matter (0.02–0.05%). They are also capable of chemoorganoheterotrophic growth during several or continuous transfers^9,10^. *Sulfobacillus* isolates use yeast extract and glucose as carbon and energy sources under mixotrophic and heterotrophic conditions^11^. They are also facultative anaerobes capable of ferric iron respiration^12–15^.

The genus *Sulfobacillus* currently includes three moderately thermophilic species and one subspecies: *S. thermosulfidooxidans*^16^, *S. thermosulfidooxidans* subsp. *asporogenes*^17^, *S. acidophilus*^18^, and *S. sibiricus*^19^. *S. thermotolerans*^20^ and *S. benefaciens*^13^ are two known thermotolerant species. Two genomes of *S. acidophilus*^21,22^ and five genomes of *S. thermosulfidooxidans*^23–25^ have been previously reported. Five more genomes assigned to *S. thermosulfidooxidans*, *S. benefaciens*, and *S. acidophilus* have been discussed^26^. In total, nine genomes of *S. thermosulfidooxidans*, two genomes of *S. benefaciens*, three genomes of *S. acidophilus*, and two genomes of *Sulfobacillus* spp. are available in the GenBank databases. Plasmid sequences of *S. thermotolerans* L15 and Y0017 have been published and analyzed^27^.

The object of the present research is *S. thermotolerans*, which is applied in bioleaching/ biooxidation of sulfidic ores and ore concentrates, containing precious (gold and silver) and non-ferrous metals. This thermotolerant species is of high industrial importance in the biotechnologies for sulfidic raw material processing at temperatures above 35 °C in the presence of elevated concentrations of toxic components^2,20,28–32^. *S. thermotolerans* proved to be resistant to high ambient concentrations of Zn^2+^ (>765 mM), Cu^2+^ (>80 mM), and Pb^2+^ (>2 mM)^33^. For *S. thermosulfidooxidans*, the minimal inhibitory concentrations (MIC) of Cu^2+^, Ni^2+^, and Zn^2+^, respectively, were >230, 292, and >800 mM^34^. In contrast to *Sulfobacillus* species, MIC of Cu^2+^, Cd^2+^, Ni^2+^, and Zn^2+^ for metal tolerant *Cupriavidus metallidurans* and *Escherichia (E.) coli* were 3–13 mM^35^ and 0.5–4 mM^36,37^, respectively.

Our research aims at providing new insights into the metabolic pathways and resistance mechanisms in bacteria of the genus *Sulfobacillus* and ACM in general. Here we present the first complete genome of the species *S. thermotolerans* and its comparison to other *Sulfobacillus* genomes. In addition to genome analysis, this work describes phenotypic and phylogenetic characteristics of *Sulfobacillus* species. We focus on the features that have been previously unknown in the genus *Sulfobacillus*, differentiate *S. thermotolerans* from other *Sulfobacillus* organisms or are unusual for acidophilic chemolithotrophs.

## Results

### General description of *S. thermotolerans* Kr1 genome

**Figure 1:**
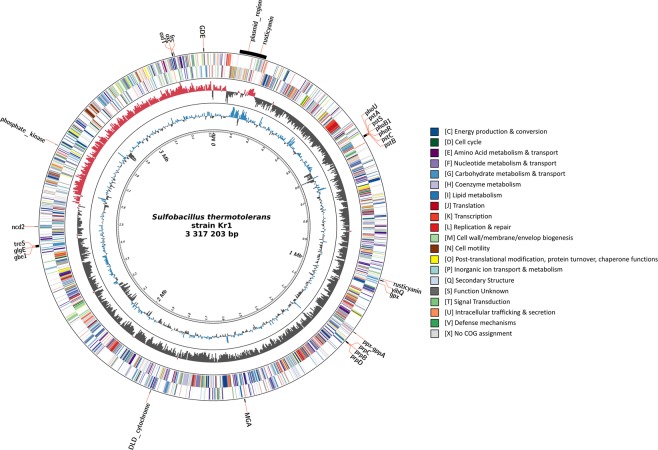
Circular map of the *Sulfobacillus thermotolerans* Kr1 genome. Rings from the outside in: (1) genes of interest and plasmid region; (2) protein-coding genes on the forward strand (color-coded by the functional categories, see legend); (3) protein-coding genes on the reverse strand (color-coded by the functional categories, see legend); (4) GC skew; (5) G + C content; (6) scale marks. Abbreviations of the genes and relevant proteins: GDE, glycogen debranching enzyme [EC 3.2.1.133]; phoU, phosphate transport system protein; pstA, phosphate transport system permease protein; pstS, phosphate transport system substrate-binding protein; phoB1, phosphatase synthesis response regulator PhoP; phoR, phosphate regulon sensor histidine kinase PhoR [EC:2.7.13.3]; pstC, phosphate transport system permease protein; pstB, phosphate transport system ATP-binding protein; yihQ, alpha-glucosidase [EC 3.2.1.20]; gpx, glutathione peroxidase [EC:1.11.1.9]; ppx-gppA, exopolyphosphatase/guanosine-5′-triphosphate,3′-diphosphate pyrophosphatase [EC 3.6.1.11 3.6.1.40]; prpC, 2-methylcitrate synthase [EC 2.3.3.5]; prpB, methylisocitrate lyase [EC 4.1.3.30]; prpD, 2-methylcitrate dehydratase [EC 4.2.1.79]; MGA, glucoamylase [EC 3.2.1.3]; DLD_cytochrome, D-lactate dehydrogenase (cytochrome) [EC 1.1.2.4]; treS, maltose alpha-D-glucosyltransferase/alpha-amylase [EC 5.4.99.16 3.2.1.1]; glgE, starch synthase (maltosyl-transferring) [EC 2.4.99.16]; gbe1; 1,4-alpha-glucan branching enzyme [EC 2.4.1.18]; ncd2, nitronate monooxygenase [EC 1.13.12.16].

**Figure 2:**
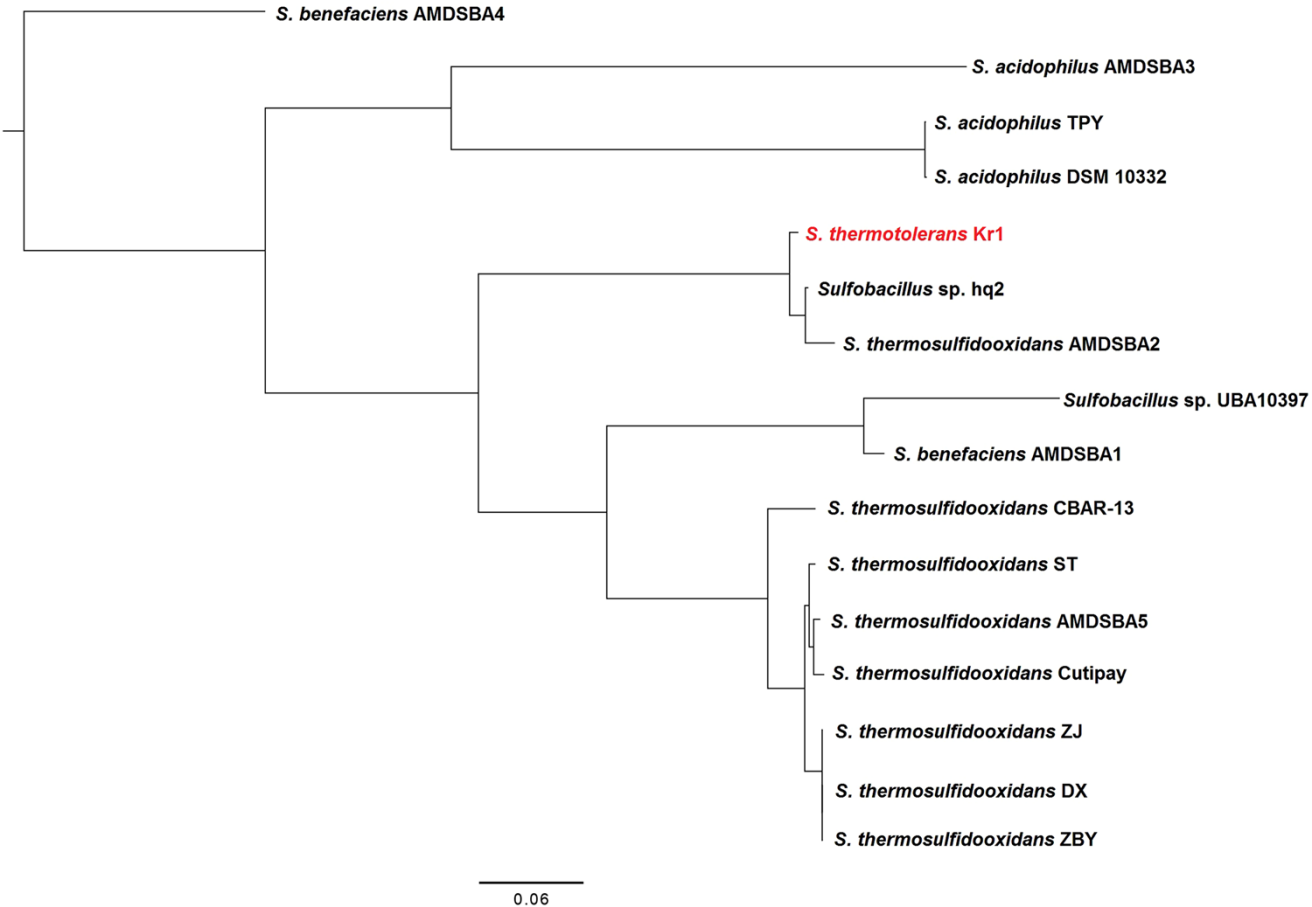
Phylogenetic position of *Sulfobacillus thermotolerans* Kr1 among other strains of the genus *Sulfobacillus*. The phylogenetic tree was constructed using the STAG (species tree inferred from all orthogroups) method (https://github.com/davidemms/STAG; OrthoFinder software package^99^) and visualized with FigTree v.1.4.3 (http://tree.bio.ed.ac.uk/software/figtree/). The tree is a consensus species tree from trees from all orthogroups (in which all *Sulfobacillus* strains were present).

The genome of *S. thermotolerans* Kr1^T^ consists of one circular chromosome (3 317 203 bp) with an overall G + C content of 52.4% (Fig. 1, Table 1). A total of 3239 genes were predicted. Among them, 3121 protein-coding genes, 67 non-translated RNAs, and 51 pseudogenes were identified. Genes with unclear functions were annotated as hypothetical proteins. Analysis of gene ontology and functional annotation of genes were carried out. The genome of *S. thermotolerans* Kr1 was compared to all available *Sulfobacillus* genomes to reveal species-specific and strain-specific features of *S. thermotolerans* Kr1. A total of 80 genes (proteins) turned out to be unique in the strain Kr1 (Table S1). Among them, we identified possible plasmid, transposon, and phage integrations, as well as MFS transporters, zinc metalloprotease, putative *c*-type cytochrome, components of the lactose/galactose catabolism, and a large number of hypothetical proteins of unknown functions. Figure 2 shows the phylogenetic position of *S. thermotolerans* Kr1 among *Sulfobacillus* strains. The phylogenetic tree is a consensus tree inferred from all orthogroups and constructed using the STAG (Species Tree Inference from All Genes) method. *Sulfobacillus* sp. hq2 proved to be the closest phylogenetic relative of the type strain Kr1.

**Table 1:** Statistics of the genome of *Sulfobacillus thermotolerans* Kr1.

Attribute	Value	% of Total
Genome size (bp)	3317203	100.00
DNA Coding region (bp)	2842887	85.70
DNA G + C content (bp)	1738272	52.40
Number of replicons	2	
Extrachromosomal elements	0	
Total genes	3239	100.00
RNA genes	67	2.06
rRNA operons	6	
Protein-coding genes	3121	96.36
Pseudo genes	51	1.57
Genes with function prediction	2716	83.85
Genes in paralog clusters	522	16.72
Genes assigned to COGs	2175	67.15
Genes assigned Pfam domains	2393	73.88
Genes with signal peptides	83	2.56
Genes with transmembrane helices	842	25.99
CRISPR repeats	4 (6)

### Identification and characterization of the plasmid-like region integrated into the chromosome of *S. thermotolerans* Kr1

**Figure 3:**
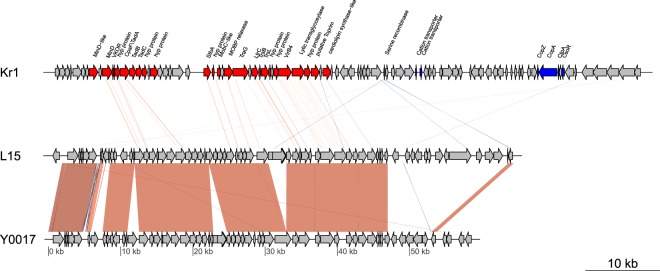
The plasmid-like region of the *Sulfobacillus thermotolerans* Kr1 genome aligned with the plasmid sequences of *S. thermotolerans* L15 and *S. thermotolerans* Y0017. The genes on the *S. thermotolerans* Kr1 chromosome, homologous to the genes of *S. thermotolerans* L15 and Y0017 plasmids, are colored red. Homologs involved in metal resistance are colored blue. The red bars connecting the plasmid genes represent direct orthologous matches. The blue bars represent reversed matches. Darker colors correspond to higher blast scores. Table S2 contains detailed information on the homology levels of the genes. The regions of high homology between the plasmids pL15 and pY0017 are colored red. Abbreviations: VKOR, vitamin K epoxide reductase; hyp protein, hypothetical protein; MOBP relaxase, MOB_P_-type family relaxase.

No extrachromosomal plasmids were identified in the strain Kr1, although each of the closest phylogenetic relatives, *S. thermotolerans* L15 and Y0017, carried cryptic plasmids (65 903 and 59 212 bp, respectively) with 63.5% overall nucleotide sequence similarity between them^27^. However, we identified a putative 83 269-bp plasmid region (ORFs BXT84_00415–00865) integrated into the chromosome of the strain Kr1 (Figs 1, 3 and Table S2). The G + C content of this region was 56–58%, which was higher than 52.4% G + C content of the chromosomal DNA (Fig. S1, Table 1).

Out of 46 ORFs located within a 40 944-bp fragment of the plasmid region, 26 of them showed close homology to the plasmid sequences of *S. thermotolerans* L15 and Y0017 (Figs 3, S2 and Table S2). A common “backbone” region coding for a probable plasmid stability system and nonpheromone conjugation system containing homologs of both type IV and II secretion systems (T4SS and T2SS) of pL15 and pY0017^27^ was identified. The genes of the common backbone region encode the MinD-like protein, CpaF-like (TadA) Flp pilus assembly ATPase-like proteins and TadB/TadC-like proteins (putative T2SS), StbA family protein (plasmid stability), relaxase, TraG-like coupling protein (a conjugation protein involved in T4SS), LtrC-like protein (primase), TrsL/TrsB putative conjugation proteins, VirB4-like protein (T4SS), and putative lytic transglycosylase (Table S2). The pL15 and pY0017 plasmids and a 40 944-bp fragment of the identified plasmid region of the strain Kr1 share accessory genes and the genes encoding eight hypothetical proteins (Fig. 3, Table S2). They include hypothetical vitamin K epoxide reductase, the phosphatidylserine/phosphatidylglycerophosphate/cardiolipin synthase-like protein, and two hypothetical proteins (ORFs BXT84_00480 and BXT84_00500) unique for the genus *Sulfobacillus* (28 and 26% a. a. identity to the cytochrome *c*-type biogenesis protein of *Myxococcus* sp. and cytochrome *c*-type biogenesis protein CcmF of *Glaciecola arctica*, respectively). The Kr1 plasmid-like region harbors a transposon containing the transposase gene (ORF BXT84_00675) with the closest similarity to *Desulforudis audaxviator* and *Desulfosporosinus acidophilus* transposases. Resolvases of the strain Kr1 are homologous to the resolvase-like proteins of pL15 and pY0017.

At the same time, the determination of possible integrations into the chromosome of *S. thermotolerans* Kr1 revealed a putative integrative and conjugation element (ICE with T4SS) within the candidate plasmid-like region (ORFs BXT84_00415–00770). However, in addition to the features of the ICE, this integrated element contained the genes specific for plasmids and the backbone and accessory genes similar to those of pL15 and pY0017 (Table S2). We, therefore, suggest that this region of the Kr1 chromosome was derived from the conjugative plasmid, which could be inserted into the genome as a result of the transposition of the Tn3-like mobile element (IS5; ORF BXT84_00675). Similarly, the genome of *S. acidophilus* TPY contains the genes of *S. acidophilus* NAL plasmids, integrated into two sites of the chromosome^22^.

We also revealed other probable gene integrations upstream of the region of homology between pL15, pY0017, and the plasmid-like region of the strain Kr1. Tn3 (ORF BXT84_00730), a transposase (ORF BXT84_00780) similar to the transposase of *Rhodopirellula baltica*, and Tn7 (ORFs BXT84_00850–BXT84_00865) could mediate them. Many genes encoded by the putative plasmid region of *S. thermotolerans* Kr1 are non-related to any genes of sequenced *Sulfobacillus* genomes (Tables S1 and S2). They are homologous to the genes of phylogenetically distinct microorganisms and, therefore, were likely acquired by horizontal gene transfer (HGT).

Interestingly, the plasmid-like region of *S. thermotolerans* Kr1 carries genetic determinants of resistance to heavy metals. These are a cation transporter homologous to that of *Acidithiobacillus (At.) ferrooxidans* and a protein homolog of the Co/Zn/Cd transporter (cation diffusion facilitator family) of *Alicyclobacillus (Al.) macrosporangiidus*. The putative integrated plasmid also codes for a copper chaperone (CopZ), two copper-translocating P-type ATPases (CopA and CtpA), and a copper-sensitive operon repressor (CsoR) (Fig. 3, Table S2). Moreover, the plasmid-like region encodes rusticyanin and a hypothetical protein homologous to the conserved hypothetical electron transporter (rnfE) of *Thiolapillus brandeum* (41% similarity; 82% coverage), which may be components of electron transport chains.

### Specific characteristics of carbon and energy metabolism

#### TCA cycle

We identified all the genes encoding the TCA cycle enzymes, including 2-oxoglutarate dehydrogenase (OGDH) complex, in *S. thermotolerans* Kr1 (Table S3). OGDH activity was measured in cell-free extracts of *S. thermotolerans* Kr1 grown at optimal (40 °C) and limiting (12 and 55 °C) growth temperatures under mixotrophic conditions. The OGDH protein possessed no detectable enzymatic activity at optimal and suboptimal (12 °C) temperatures. However, an increase in the growth temperature from 40 to 55 °C induced OGDH activity from zero to 6.7–6.9 nmol/min mg protein (enzyme assays were carried out in three replicates).

#### Glyoxylate and methylcitrate cycles

**Figure 4:**
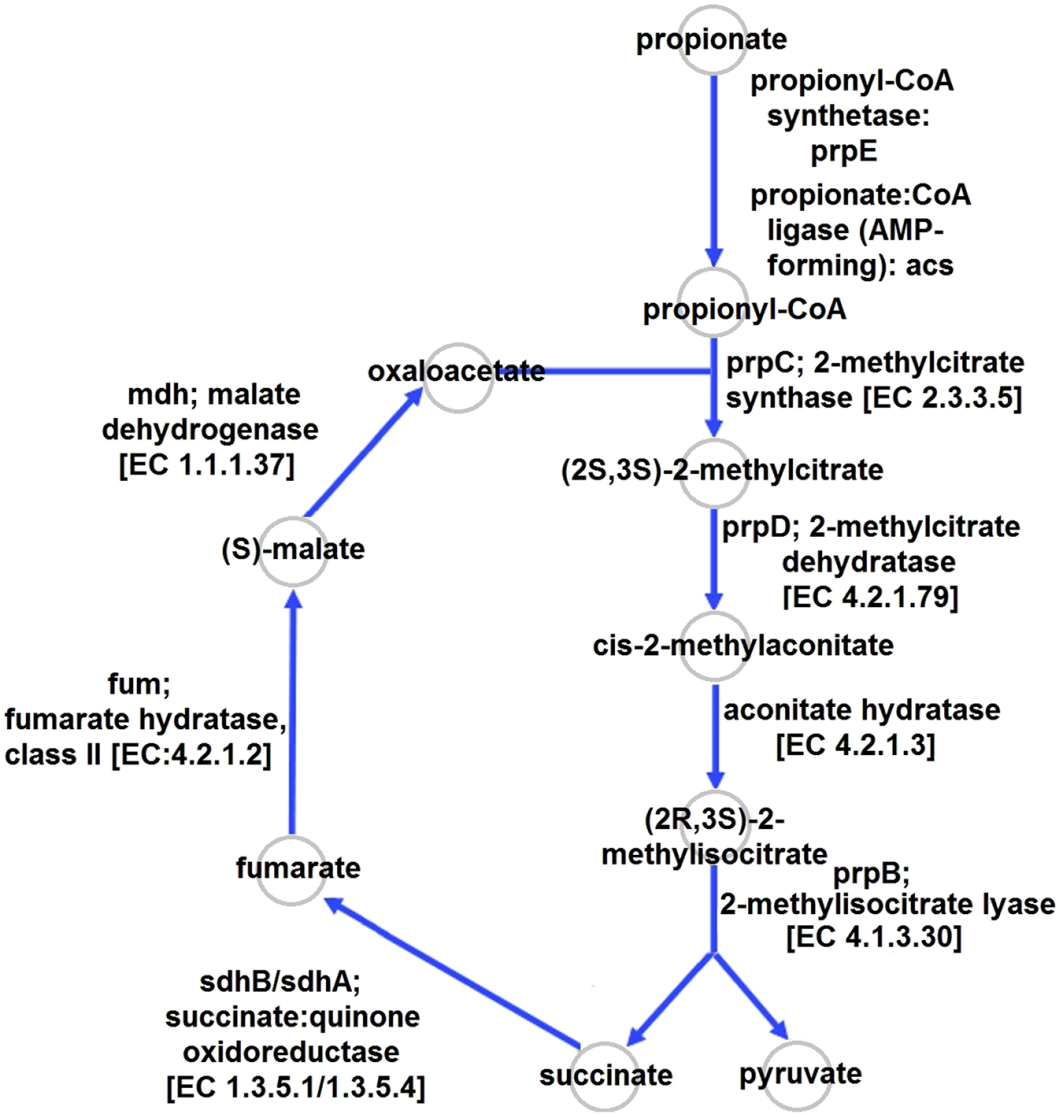
The methylcitrate cycle predicted in *Sulfobacillus thermotolerans* Kr1. Arrows indicate reactions catalyzed by the enzymes encoded in the genome.

The glyoxylate cycle of *S. thermotolerans* Kr1 is incomplete due to the absence of the isocitrate lyase gene, although the malate synthase gene is present in the genome of this strain (Table S3). Nevertheless, genome analysis revealed the genes encoding the methylcitrate cycle in *S. thermotolerans* Kr1 (Fig. 4, Table S4). The key methylcitrate cycle enzymes—2-methylcitrate synthase (MCS), 2-methylcitrate dehydratase, and methylisocitrate lyase (MCL)—were identified. The comparison of the genome of the strain Kr1 to other *Sulfobacillus* genomes indicated the genes encoding these enzymes only in *S. thermosulfidooxidans*, *S. benefaciens*, and the strain hq2. The key methylcitrate cycle enzymes of these species share 73–99.5% a. a. identity (97–100% coverage). MCS activity in the extracts of the Kr1 cells grown in the medium containing propionate (0.02–0.08%) was shown to increase with an increase in propionate content in the cultivation medium. The values of MCS activity were close in the presence of 0.02–0.04% propionate: 30.9–34.2 units/min mg protein. When concentrations of propionate were increased to 0.06 and 0.08%, MCL activities reached 51.8–55.4 and 70.2–72.5 units/min mg protein, respectively. At the same time, the maximal cell yields decreased (2.1–2.2 and 1.5–1.8 × 10^8^ cells/ml in the presence of 0.02–0.04 and 0.06–0.08% propionate, respectively) in comparison with the optimal mixotrophic variant (3.5 × 10^8^ cells/ml).

#### Oxalate degradation

The gene cluster coding for the formyl-CoA transferase (Frc) and oxalyl-CoA decarboxylase (Oxc) enzymes and the oxalate: formate antiport protein (OxlT) gene were identified in *S. thermotolerans* Kr1 (Table S5). These proteins are involved in the oxalate degradation pathway. The genomic comparisons between the members of the genus *Sulfobacillus* indicated that *S. thermotolerans*, *Sulfobacillus* sp. hq2, *S. benefaciens*, and *S. acidophilus* harbored all three proteins of this pathway. The level of identity (a. a.) between the proteins of *S. thermotolerans* Kr1 and other *Sulfobacillus* species was within 72‒100% (95‒100% coverage).

#### Lactose and galactose catabolism

The components of the lactose–galactose catabolism pathway were identified in the *S. thermotolerans* Kr1 genome. It codes for α- and β-galactosidases, as well as other proteins of lactose/galactose catabolism (transporters and Lac I family transcriptional regulator, in particular) (Table S1). No other *Sulfobacillus* genomes encode these proteins. Most of the closest protein homologs belong to bacteria of the genus *Alicyclobacillus*, which are also members of the communities of ACM. The closest homologs of α- and β-galactosidases of the strain Kr1 belong to *Paenibacillus* and *Alicyclobacillus* spp. (52–57% similarity; 94–99% coverage) and *Thermoanaerobacter* and *Alicyclobacillus* spp. (46–47.1% similarity; 96–98% coverage), respectively. The Lac I family transcriptional regulator was most similar to that of *Alicyclobacillus* spp. (47.2–48.3% similarity; 96–97% coverage). The closest homologs of the carbohydrate ABC transporter permease, lactose ABC transporter permease, and sugar ABC transporter substrate-binding protein belonged to *Alicyclobacillus* spp. as well: 61.2–65.9% similarity (99–100% coverage), 62.2–67.2% similarity (97–98% coverage), and 49.6–64.2% similarity (91–98% coverage), respectively.

#### Ferrous iron oxidation and electron transfer chain

**Figure 5:**
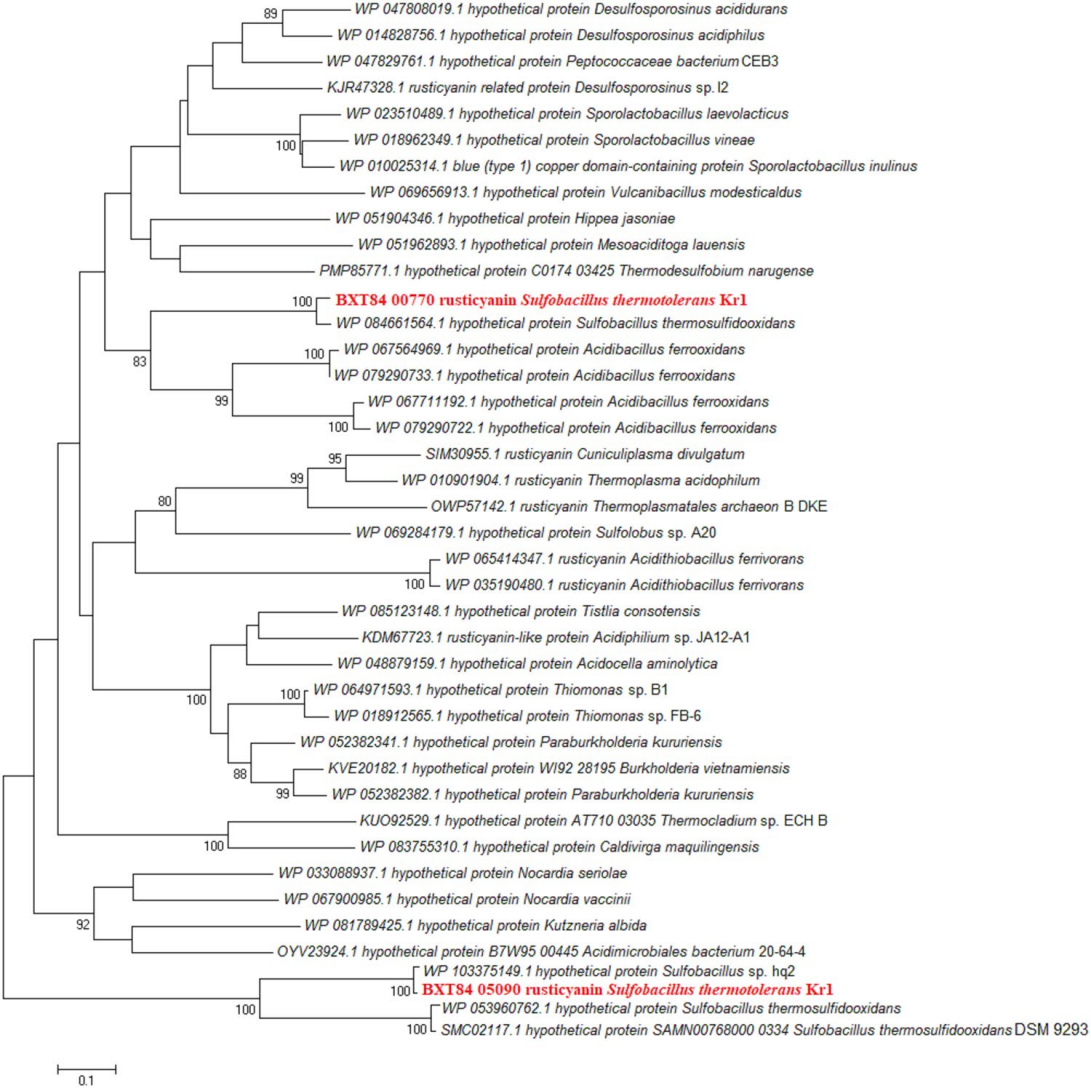
Phylogenetic positions of the rusticyanin proteins of *Sulfobacillus thermotolerans* Kr1. The phylogenetic tree was constructed in MEGA7^109^. The evolutionary history was inferred using the neighbor-joining method^110^. The evolutionary distances were computed using the Poisson correction method^112^. The analysis involved 40 amino acid sequences. All positions containing gaps and missing data were eliminated. There were a total of 87 positions in the final dataset. The percentage of replicate trees, in which the associated taxa clustered together in the bootstrap test (1000 replicates), are shown next to the branches^111^ (values lower than 80% are hidden).

Table S6 shows electron transfer components encoded in the genome of *S. thermotolerans* Kr1. Interestingly, we identified two genes coding for rusticyanins. The results of alignment indicated a 30% a. a. similarity between these rusticyanins (Fig. S3). A comparison of their amino acid sequences to protein databases and subsequent phylogenetic analysis showed the following. Apart from the hypothetical protein of *S. thermosulfidooxidans* 9293 (96% a. a. similarity; 100% coverage), the proteins of *Acidibacillus ferrooxidans* proved to be the closest homologs of the rusticyanin-like protein of the strain Kr1 (42–43% a. a. similarity; 62–68% coverage). Thus, the rusticyanin-like proteins of *S. thermotolerans* Kr1 and *S. thermosulfidooxidans* 9293 occupy a common branch in the phylogenetic tree (Fig. 5). Another rusticyanin of the strain Kr1, together with the closest rusticyanin homolog of *Sulfobacillus* sp. hq2 (99.3% similarity; 100% coverage), shows a 53–54% a. a. similarity (100% coverage) to *S. thermosulfidooxidans* proteins. Therefore, rusticyanins of these *Sulfobacillus* species form a common cluster, which consists of two separate branches (Fig. 5). The rusticyanin-related protein of *Desulfosporosinus* sp. is the next closest protein to this rusticyanin of the strain Kr1 (35% a. a. identity; 80% coverage).

Three genes of *S. thermotolerans* Kr1 were annotated as sulfocyanins with the closest homology to the sulfocyanin proteins of the strain hq2 and *S. benefaciens*. The genome of the strain Kr1 also encodes three cytochromes *c* homologous to the proteins of other bacteria of the genus *Sulfobacillus*.

While the *bc*_1_ complex was not identified in the strain Kr1, cytochrome *b* subunit of the *bc1* complex (2 copies) and NADH dehydrogenase (14 subunits and F-type ATPase) were present (Table S6). *S. thermotolerans* Kr1 contained the genes coding for three terminal oxidase complexes. Components of the cytochrome *c* oxidase (three clusters of the *coxBAC* genes), cytochrome *aa*_3_ menaquinol oxidase (*qoxABCD*), and three copies of the cytochrome *bd* ubiquinol oxidase were identified (Table S6).

### Stress resistance and defense systems

#### Oxidative stress defense

We revealed several enzymes, which were associated with defense mechanisms against reactive oxygen species in the strain Kr1 (Table S7). Glutathione (GSH) peroxidase was found only in the Kr1 and hq2 strains. The closest homologs of the GSH peroxidase of *S. thermotolerans* belong to *Al. macrosporangiidus* and *Aphanothece hegewaldii*. The genome of the strain Kr1 harbors superoxide dismutase (SOD) and several peroxiredoxins/alkyl hydroperoxide reductases, which are also present in *S. thermosulfidooxidans* and *S. acidophilus* genomes^26^, as well as in the strain hq2 and *S. benefaciens* (this study). Atypical 2-cys peroxiredoxin was revealed in *S. thermotolerans* Kr1, *Sulfobacillus* sp. hq2, and two strains of *S. acidophilus* and *S. thermosulfidooxidans*. GSH peroxidase and SOD possessed activities of 4.4–4.6 units/min mg protein in cell-free extracts of *S. thermotolerans* Kr1 grown at partial pressure of atmospheric oxygen. When *S. thermotolerans* cells were subsequently cultivated at intense aeration, activities of antioxidant enzymes increased from 4.5–4.6 to 36.9–38.9 units/min mg protein (GSH peroxidase) and from 4.4–4.5 to 57.0–61.2 units/min mg protein (SOD). Thus, activities of these two enzymes of the oxidative stress defense were up-regulated in response to active aeration.

#### Resistance to heavy metals and metalloids

Apart from the metal resistance determinants localized within the putative chromosome integrated plasmid, the genome of *S. thermotolerans* Kr1 encodes other metal resistance systems. Similarly to other species of the genus *Sulfobacillus*, metal efflux, which involves metal ion efflux proteins^24^, can be an essential mechanism of defense against heavy metals in the cells of *S. thermotolerans* Kr1. Thus, metal efflux can occur *via* transport systems composed of the ATP-binding cassette (ABC) superfamily and major facilitator superfamily (MFS) proteins, as well as cation translocating P-type ATPases (Table S7). The genome of *S. thermotolerans* Kr1 encodes another resistance determinant: the MerA mercuric reductase. The genes participating in the mechanisms of resistance to arsenic were identified in *S. thermotolerans* Kr1 as well (Table S7). Although the repressor of the arsenic resistance operon (ArsR) was present in *S. thermotolerans* Kr1, no arsenate reductase (ArsC) was identified. *S. thermosulfidooxidans* strains Cutipay^23^ and ST^24^ contained the *arsC* and *arsR* genes. The two-gene *ars* operon is speculated to give resistance to only trivalent metalloid salts of As, whereas ArsC is required for arsenate resistance^38^. At the same time, the ArsA/ArsB ATPase pump predicted to export arsenite and antimonite^39^ was found in *S. thermotolerans* Kr1 and all other sequenced *Sulfobacillus* genomes.

**Figure 6:**
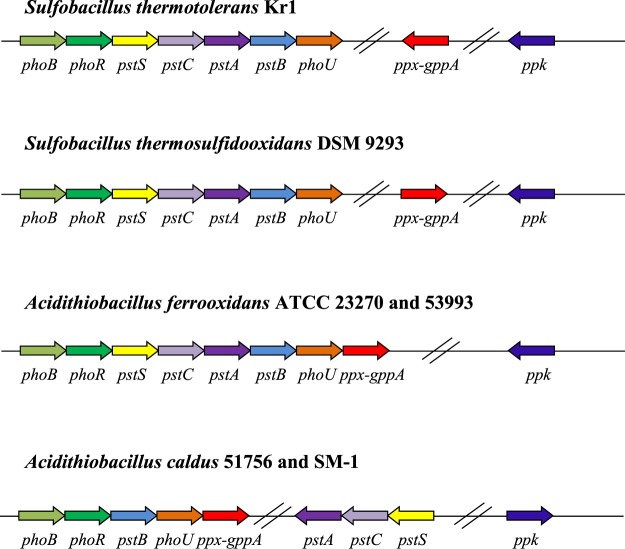
Putative *Pho* regulon and the polyphosphate kinase and exopolyphosphatase genes, involved in the polyphosphate-based mechanism of metal resistance in *Sulfobacillus thermotolerans* Kr1 and *S. thermosulfidooxidans* DSM 9293 (the genome sequence accession number NZ_FWWY00001), in comparison to those of *Acidithiobacillus ferrooxidans* and *At. caldus* strains^41^. Abbreviations of the genes encoding the corresponding proteins: *phoB*, phosphatase synthesis response regulator PhoP; *phoR*, phosphate regulon sensor histidine kinase PhoR; *pstS*, phosphate transport system substrate-binding protein; *pstC*, phosphate transport system permease protein; *pstA*, phosphate transport system permease protein; *pstB*, phosphate transport system ATP-binding protein; *phoU*, phosphate transport system protein; *ppx-gppA*, exopolyphosphatase/guanosine-5′-triphosphate,3′-diphosphate pyrophosphatase; *ppk*, polyphosphate kinase.

For the first time, we identified a putative *Pho* regulon consisting of *phoB, phoR, pstS, pstC, pstA, pstB*, and *phoU*, as well as the *ppx* and *ppk* genes, in *S. thermotolerans* Kr1 and bacteria of the genus *Sulfobacillus* in general (Fig. 6, Table S7). These genes are involved in the polyP-based mechanism of resistance to heavy metals. The comparison of the genome of *S. thermotolerans* Kr1 to other *Sulfobacillus* genomes available in the databases revealed a putative polyP-based resistance mechanism in *S. thermosulfidooxidans* as well: 61–62 and 70% a. a. identity for PPK and PPX, respectively, and 76–100% a. a. identity for the proteins encoded by the *Pho* regulon. We also identified the *Pho* regulon genes in *S. acidophilus* genomes, which, however, lacked the *ppk* and *ppx* genes.

## Discussion

Some ACM, including members of the genus *Sulfobacillus*, were shown to withstand extremely high concentrations of heavy metals in the environment^33,34,40,41^. Heavy metals and metalloids reach high concentrations in the mine drainage waters, industrial bioleaching tanks, mine deposits, and sulfide ore heaps. *S. thermotolerans* Kr1 is tolerant to high concentrations of zinc, copper and other heavy metals (more than 1–2 orders of magnitude higher in comparison to the majority of the known organisms)^33^. Extremely high resistance of *S. thermotolerans* Kr1 to heavy metals is of particular interest since communities of ACM resistant to high concentrations of metals are essential for the efficient microbial stage of oxidation in industrial processes. Analysis of the first sequenced genome of *S. thermotolerans*, together with other results reported in the current paper, provided new insights into resistance capacities of this species.

In general, the exact mechanisms of extreme resistance in ACM are still not evident^41^. Analyses of the ACM genomes revealed metal resistance determinants varying in content and quantity. Acidophilic chemolithotrophs harbor metal resistance systems responsible for import and efflux of heavy metal ions, as well as their extra- and intracellular sequestration and transformation to less toxic compounds^24,41–44^. At the same time, the presence of an increased number of metal resistance genes has not been shown to increase resistance in acidophiles^45^. High metal resistance was found to be associated with complexation of free metals by sulfate ions and passive tolerance to metal influx via an internal positive cytoplasmic transmembrane potential^45^.

The study of the expression of the genes determining resistance to copper in *At. ferrooxidans*, together with comparative proteomics, revealed regulation of the copper resistance genes and proteins in response to Cu. Thus, RND-type Cus systems and different RND-type efflux pumps were up-regulated, while the proteins involved in the influx of cations into the cell were down-regulated^41,43,46–50^. Analysis of transcriptional expression in the cells of *Ferroplasma (F.) acidarmanus* and *Sulfolobus (Sl.) metallicus* archaea, exposed to high copper, indicated increased transcript levels of several copper resistance determinants. At the same time, the proteomic study of these archaea revealed only the regulation of protein expression associated with energy production and conversion, biosynthesis of amino acids, and general stress response^44,51^. No regulation of expression of the components of the zinc resistance system was revealed in the cells of *At. caldus* and *Acidimicrobium (Am.) ferrooxidans* in response to zinc either, while an increased synthesis of the efflux Zn transporter and several hypothetical proteins of unknown functions was observed in *F. acidarmanus*^52^. However, the published data do not provide a complete understanding of the phenomenon of extreme metal resistance, which can involve unknown mechanisms of defense, and give no information about the mechanisms of metal resistance in the genus *Sulfobacillus*.

Our data indicate that apart from the identified metal resistance determinants common for ACM and listed above and in Table S7, *S. thermotolerans* probably uses an additional mechanism for metal resistance, which has not been previously known in Gram-positive ACM (Fig. 6). This mechanism is based on the metal ion sequestration with polymers of inorganic polyphosphate (polyP) and has been reported in several acidophilic archaea and Gram-negative bacteria. Thus, a polyP-dependent copper resistance mechanism was proposed for polyP-accumulating acidophiles: *At. ferrooxidans*^53^, *Sulfolobus metallicus*^54^, *Metallosphaera sedula*, *At. thiooxidans*^43,55,56^, and *At. caldus*^52^. The polyphosphate kinase (PPK) enzyme catalyzes the reversible conversion of the terminal phosphate of ATP into polyP, which is hydrolyzed to inorganic phosphate by exopolyphosphatase (PPX). Inorganic phosphate, in turn, binds metal cations forming a metal-phosphate complex transported out of the cell by phosphate transporters. In *E. coli*, the *ppk* and *ppx* genes belong to the same operon and are co-regulated at the transcriptional level. Thus, accumulation of polyP granules is not observed in *E. coli*, since the PPK and PPX catalyze opposite reactions^57^. In *At. ferrooxidans* and *At. caldus*, the *ppk* and *ppx* genes are located in different operons (Fig. 6), which implicates separate regulation of polyP synthesis and degradation^41,58^. These *Acidithiobacillus* isolates were also shown to contain putative *Pho* regulons different from each other and from the *Pho* regulons of other bacteria^41,52,58^. The genetic organization of the components of the polyP mechanism in *S. thermotolerans* Kr1 turned out to be different from those of the known ACM. Thus, in contrast to acidithiobacilli, the PPX and PPK of the strain Kr1 were separated from the *pho* and *pst* genes and did not belong to the same operon either (Fig. 6). In our previous paper, we showed the presence of polyphosphate granules in the cells of *S. thermotolerans* Kr1^29^. A comparison to other *Sulfobacillus* genomes revealed that the type strain *S. thermosulfidooxidans* 9293, which is also highly resistant to heavy metals^34,40,43^, harbored all the components for the polyP-dependent mechanism as well (Fig. 6). These two *Sulfobacillus* species, which are highly resistant to metals (in contrast to *S. acidophilus* and *S. benefaciens* that are tolerant to lower metal content in the environment^13,33,34,40^), together with the ACM listed above, seem to possess the polyP-dependent mechanism, which can also contribute to the extreme metal tolerance.

Interestingly, *S. thermotolerans* Kr1 carried genetic determinants of heavy metal resistance, which probably belonged to the putative chromosome integrated conjugative plasmid containing homologs of both type IV and II secretion systems (T4SS and T2SS) (Fig. 3, Table S2). A putative integrative and conjugation element (ICE) with T4SS, identified within this region, was probably integrated into the chromosome of the Kr1 strain as a part of the plasmid or separately from other integrations that are located upstream of the ICE region. It should be noted that most ACM harbor cryptic plasmids, which do not contribute to the phenotype of the host cells. Localization of heavy metal resistance determinants on plasmids or integrative and conjugative elements is not typical for acidophilic chemolithotrophs. These genes are primarily located on the chromosomes outside mobile element regions or integrated extrachromosomal elements. The determinants of copper resistance, located within the genomic island of the Gram-negative bacterium *At. ferrooxidans* ATCC 53993^47^, and determinants of zinc resistance, localized on the megaplasmid of *At. caldus* SM-1^59^, are exceptions reported to date. The copper resistance genes are also suggested to be present within the metagenomic island in the *At. ferrivorans* genome^60^.

The functions of the metal resistance determinants identified within the integrated plasmid of *S. thermotolerans* Kr1 are associated with the tolerance to copper and some other bivalent cations of heavy metals. The repressor encoded on the plasmid of the strain Kr1 responds to stressors, including Cu^2+^, Zn^2+^, Ag^+^, Cd^2+^, and Ni^2+^ ions^61^, while P-type ATPases and copper chaperones transfer metal ions such as Cu^2+^, Cd^2+^, Co^2+^, and Zn^2+ ^^62,63^. However, while the *copA*, *copZ*, and *csoR* genes are also localized on the chromosome of *S. thermotolerans* Kr1, other metal resistance determinants of the integrated plasmid are present only within the plasmid region. The involvement of the rusticyanin gene localized in the vicinity of these metal determinants in metal resistance mechanisms is also not excluded. Thus, the CopZ-like protein and periplasmic rusticyanin of *At. ferrooxidans* ATCC 23270 contributed to its high-level copper resistance^64^. In this bacterium, copper cupredoxins (rusticyanin, in particular) not only function as components of the ferrous iron oxidation pathway but also may bind excess copper in the periplasm, most likely playing a role in high copper resistance^64^.

Our previous study of the polymorphism of plasmid profiles in different *Sulfobacillus* strains revealed alterations in the number of *S. thermotolerans* Kr1 plasmids (from one to three) depending on the presence of heavy metals in the cultivation media. The variant grown in the medium with high concentrations of zinc contained a single plasmid^33^. This may indicate that the stable integration of the plasmid into the chromosome was relatively recent during the period when the strain Kr1 was maintained in our laboratory. Localization of the metal resistance determinants on plasmids can be advantageous under the conditions of extremely high concentrations of heavy metals in the natural and technogenic habitats specific for ACM.

Our current research revealed new data on the carbon metabolism in bacteria of the genus *Sulfobacillus* as well. Although all the genes encoding the TCA cycle enzymes were identified in all *Sulfobacillus* genomes, the TCA cycle was supposed to be incomplete due to the lack of 2-oxoglutarate dehydrogenase (OGDH) activity^9^. The cell-free extracts of *S. thermotolerans* Kr1 grown under mixotrophic, organotrophic or autotrophic conditions lacked OGDH activity^29^, similarly to *S. sibiricus*^65^, *S. thermosulfidooxidans*, and *S. thermosulfidooxidans* subsp. *asporogenes*^9^. We detected no OGDH activity in the cell-free extracts of *S. thermosulfidooxidans* and *S. thermosulfidooxidans* subsp. *asporogenes*, grown in the presence of high zinc (0.8 M Zn^2+^) in the medium or low ambient pH 1.2^66^_._ The results obtained in the present study confirmed our previous data on zero OGDH activity in *S. thermotolerans* Kr1 at 40 and 12–14 °C^29^. Nevertheless, the change in the temperature of cultivation from 40 to 55 °C induced OGDH activity. It was also detected in two thermophilic species of the genus *Sulfobacillus* under stress growth conditions: at the limiting growth temperature of 18 °C (*S. sibiricus* N1) and under unfavorable autotrophic conditions (*S. thermosulfidooxidans*) (our unpublished data). Thus, the incomplete TCA cycle could switch to the complete version under specific adverse conditions. The disrupted TCA cycle does not generate energy (no ATP or reductive equivalents are produced) but provides precursors for biosynthetic processes. However, when *Sulfobacillus* organisms endure stress, a complete TCA cycle fulfills its common energetic function and probably provides for a higher respiration rate required to withstand such conditions.

In the present work, we revealed only one key enzyme of the glyoxylate bypass, malate synthase, encoded in the genome of *S. thermotolerans* Kr1; the isocitrate lyase gene was not identified. The biochemical study of the cell-free extracts of the Kr1 strain indicated no isocitrate lyase activity either, irrespective of the growth temperature or nutrition type, whereas malate synthase activity was detected under all cultivation conditions^29^. *S. thermosulfidooxidans, S. thermosulfidooxidans* subsp. *asporogenes*, and *S. sibiricus* lacked the functional glyoxylate bypass either^9,65^. At the same time, the glyoxylate cycle plays a vital role in anaplerosis, converting acetyl-CoA (derived from the oxidation of both odd- and even-chain length fatty acids) into oxaloacetate (the intermediate of the citric acid cycle)^67,68^.

However, we identified the genes coding for the key enzymes of the methylcitrate cycle previously unknown in the genus *Sulfobacillus* (Fig. 4). This cycle is a modified version of the TCA cycle, and it metabolizes propionyl-CoA generated by β-oxidation of odd-chain-length fatty acids and converts it into pyruvate^69^. Our prior study revealed that cultivation of *S. thermosulfidooxidans, S. sibiricus*, and *S. thermotolerans* resulted in the accumulation of propionate and acetate in the media under the conditions of oxygen deficiency and the continuous consumption of these exometabolites during the subsequent growth^14^. In natural habitats and biotechnological processes, bacteria of the genus *Sulfobacillus* can be subjected to unfavorable conditions of decreased oxygen content (e.g., in sulfide ore deposits, thermal springs, bioleaching heaps, and zones of bioleaching tanks with poor aeration), when propionate is accumulated. The culture liquid contained propionate (0.9 mg/l) at the late-exponential phase of growth of *S. thermotolerans* at atmospheric oxygen partial pressure (without intense aeration) in the medium supplemented with Fe^2+^, yeast extract, and glucose^14^. Propionate and its derivative were also detected among the metabolites of *S. thermotolerans* Kr1, which predominated in the communities of ACM forming biofilms on the pyrite-arsenopyrite ore concentrate in the presence of yeast extract (0.02%) in percolators (our unpublished data). In the present study, we showed that *S. thermotolerans* Kr1 was capable of growth in the presence of propionate (0.02–0.08%) under mixotrophic conditions. The growth parameters decreased gradually with an increase in propionate concentration, indicating the toxic effect of the latter.

The methylcitrate pathway is predominantly found in pathogenic bacteria: *Neisseria meningitides*, *Salmonella enterica*, *E. coli*, *Mycobacterium* (*M*.) *tuberculosis*, and *M. smegmatis*^70^. These microorganisms use the methylcitrate pathway to utilize propionate for carbon and energy and/or to prevent its intracellular accumulation to toxic levels. Thus, propionate catabolism can be an alternative detoxification mechanism^71,72^. In our current study, we analyzed other *Sulfobacillus* genomes for the presence of the key enzymes of the methylcitrate cycle as well. Apart from *S. thermotolerans*, the genes encoding these enzymes were revealed in *S. thermosulfidooxidans* and *S. benefaciens*. Remarkably, we identified no genes encoding the components of the methylcitrate cycle in *S. acidophilus*. On the contrary, the complete glyoxylate cycle containing both isocitrate lyase and malate synthase was predicted only in *S. acidophilus* TPY and 10332^26^. Enzyme assays revealed an increase in 2-methylcitrate synthase activity (MCS, the key enzyme of the methylcitrate cycle) in the cell-free extracts of *S. thermotolerans* in response to increased concentrations of propionate in the medium of growth. Similar direct interrelations between the propionate concentration and MCS activity were shown for *S. thermosulfidooxidans* DSM 9293 and *S. sibiricus* N1 (our unpublished data). We suggest that propionyl-CoA conversion to pyruvate in the methylcitrate cycle and further to PEP can contribute to the replenishment of biosynthesis precursors (anaplerosis) and gluconeogenesis in the *Sulfobacillus* strains that lack anaplerotic glyoxylate cycle. The methylcitrate cycle can also be a detoxification mechanism.

In contrast to all other *Sulfobacillus* genomes, the genome of the strain Kr1 contains the genes involved in the catabolism of lactose and galactose. The closest homologs of the lactose catabolism proteins of the strain Kr1 belong to *Alicyclobacillus* species, which are also members of acidophilic chemolithotrophic communities. Therefore, we suggest that these genes were acquired by *S. thermotolerans* Kr1 as a result of HGT. It is unclear whether corresponding proteins can function in this strain. Nevertheless, β-galactosidase of *Al. acidocaldarius* proved to be thermostable, highly active and potentially useful as a commercial product for lactose hydrolysis^73^.

Ferrous iron oxidation in the *Sulfobacillus* genus is another aspect, which is of both environmental and biotechnological importance. Although the investigation of this problem started almost 30 years ago^74–78^, Fe^2+^ oxidation in bacteria of the genus *Sulfobacillus* is still poorly understood. The initial electron transfer components accepting electrons directly from Fe^2+^ remain to be elucidated.

In the present study, we identified two rusticyanin genes in *S. thermotolerans* Kr1. These rusticyanins proved to occupy separate branches in the phylogenetic tree (Fig. 5). *S. acidophilus* lacks homologs of rusticyanins typically found in iron-oxidizing acidophiles^24^. The rusticyanin proteins were annotated only in the species *S. thermosulfidooxidans* and the strain AMDSB5^26^. Rusticyanins are periplasmic blue copper proteins, which are components of electron transport chains involved in the oxidation of ferrous iron and reduced sulfur compounds in Gram-negative chemolithotrophic bacteria and some archaea^79–81^. Interestingly, the role of rusticyanins in Gram-positive ACM, which differ from Gram-negative bacteria in the cell wall structure, is unclear. We suggest that identified rusticyanins can participate in the initial stages of the ferrous iron oxidation process in *S. thermotolerans* Kr1. Rusticyanins, together with cytochromes *c*, may also be involved in Fe^3+^ respiration system^82–84^.

Sulfocyanins (the blue copper redox proteins commonly found in archaea) encoded in the genome of the strain Kr1 were previously identified in *S. thermosulfidooxidans* strains^24,25,78^. A high level of expression of sulfocyanins was registered in the cells of *S. thermosulfidooxidans* ST grown on the medium with ferrous iron^24^. These proteins belong to the archaeal cluster of sulfocyanins.

The presence of the cytochrome *c*, rusticyanin, and sulfocyanin genes in the genome of *S. thermotolerans* Kr1 may suggest that the membrane-bound cytochrome *c* directly reduced by Fe^2+^ transfers electrons to sulfocyanin, rusticyanin or other components of the transport chain. All sequenced *Sulfobacillus* genomes were shown to harbor one or few cytochrome *c* copies, except for *S. benefaciens* AMDSBA1^26^. Identification of the prosthetic cytochrome groups in the cells of *S. thermotolerans* Kr1 predicted the presence of heme *c*^29^.

The *bc*_1_ complex, functioning in reverse and encoded by the *petI* operon^85^, was not identified in the strain Kr1. Only the strain AMBDSBA4 (divergent from the known *Sulfobacillus* species) was predicted to harbor *bc* complex, which can be involved in the reverse electron transfer^26^. The genome of *S. thermotolerans* Kr1 encodes only a cytochrome *b* subunit of the *bc1* complex (2 copies) (Table S6), similarly to *S. acidophilus* and *S. thermosulfidooxidans* genomes. NADH dehydrogenase (14 subunits and F-type ATPase) is present as well. The terminal oxidase complexes (cytochrome *c* oxidase, cytochrome *aa*_3_ menaquinol oxidase, and *bd* ubiquinol oxidase) identified in *S. thermotolerans* Kr1 are probably responsible for the terminal stage of iron oxidation. *S. thermosulfidooxidans* TH1, *S. acidophilus* ALV, and *S. sibiricus* N1 possessed cytochrome *aa*_3_-type oxidase activity^74–76^, while *S. thermosulfidooxidans* DSM 9293 yielded spectra dominated by *a*-type cytochromes^77^. Cytochromes and cytochrome oxidases were upregulated during the growth of *S. thermosulfidooxidans* DSM 9293 when the chalcopyrite ore concentrate was the sole source of iron^78^.

The genes involved in oxalate degradation were revealed in bacteria of the genus *Sulfobacillus* for the first time. They were identified in *S. thermotolerans*, *S. benefaciens*, and *S. acidophilus* species. Some oxalate-degrading microorganisms are known to use oxalate as a carbon and energy source, while others do not require oxalate for growth and decompose it at concentrations toxic for cells^86–88^. The presence of these genes in the *S. thermotolerans* Kr1 genome implies high metabolic capacities of this thermotolerant species, as well as the possible detoxification mechanism against high concentrations of oxalate.

*S. thermotolerans* has developed a more complex antioxidant system in comparison to other species of the genus *Sulfobacillus*. This system consists of superoxide dismutase (SOD), several peroxiredoxins/alkyl hydroperoxide reductases, 2-cys peroxiredoxin, and glutathione (GSH) peroxidase, which reduces hydroperoxides by GSH. The genomic comparisons revealed that no other species of the genus *Sulfobacillus* harbored GSH peroxidase. At the same time, oxidative stress is one of the main factors inhibiting microbial growth and substrate oxidation under aerobic conditions^89^. Diverse redox-active metal ions (iron, copper, cobalt, and nickel) also cause oxidative stress^90^. Biotank processes of leaching of sulfide raw materials by microbial communities (including bacteria of the genus *Sulfobacillus*) are associated with intense aeration and metal accumulation in the liquid phase. Our results showed that GSH and SOD activities in *S. thermotolerans* Kr1 depended on the aeration mode and increased 8.3–13.3 times at intense aeration. Thus, the induction of the antioxidant defense enzymes, including GSH peroxidase, is one of the strategies of *S. thermotolerans* Kr1 against adverse effects of intense aeration. Since the closest homologs of the GSH peroxidase belong to phylogenetically distinct microorganisms, the GSH peroxidase gene was probably acquired by *S. thermotolerans* as a result of HGT.

This work reports the first sequenced and annotated genome of the biotechnologically significant species *S. thermotolerans* isolated from the industrial process. Analysis of its genome sequence, genomic comparisons to other *Sulfobacillus*, as well as physiological and biochemical characteristics, provide new insights into versatile metabolic pathways and resistance mechanisms in the genus *Sulfobacillus*. We suggest that the probable plasmid-mediated resistance to heavy metals, propionate metabolism, methylcitrate cycle, effective oxidative stress defense, oxalate degradation, as well as flexible carbon and energy metabolism, gave this strain a competitive advantage in the communities of ACM under unstable conditions of natural metal-rich environments and industrial bioleaching processes.

Our data on the *S. thermotolerans* Kr1 genes and proteins, which are phylogenetically distinct from those of *Sulfobacillus* strains and similar to those of other microbial taxa that inhabit the same environments, indicate genetic exchange between members of the communities of ACM. These proteins are involved in metabolic and resistance systems, which suggests that HGT played a vital role in the development of the highly resistant phenotype of *S. thermotolerans*. Future research should further develop our findings on resistance to heavy metals and toxic compounds, defense strategies against unfavorable factors, constructive metabolism, and energy processes in bacteria of the genus *Sulfobacillus*. The results will help to gain deeper understanding of ecological functions of *Sulfobacillus* organisms and interactions among members of the natural and industrial communities of acidophilic chemolithotrophs.

## Material and Methods

### Cultivation of *S. thermotolerans* Kr1

*S. thermotolerans* strain Kr1^T^ (VKM B-2339^T^ = DSM 17362^T^)^20^ was cultivated at 39 ± 1°С in 2500-ml Erlenmeyer flasks containing 1500 ml of the modified 9K medium^91^ supplemented with yeast extract (0.02%, wt/vol). The 9K medium contained the following (g/l): (NH_4_)_2_SO_4_, 3.0; KCl, 0.1; KH_2_PO_4_, 0.5; MgSO_4_ · 7H_2_O, 0.5; Ca(NO_3_)_2_ · 4H_2_O, 0.01; and FeSO_4_ · 7H_2_O, 15 g/l (36 mM Fe^2+^). The pH values and concentrations of Fe^3+^ and Fe^2+^ were measured as described^31^. The solutions of H_2_SO_4_ (10 N) and NaHCO_3_ (20%) were used to adjust the initial pH value to 1.8. The amount of inoculum was 10% (vol/vol).

*S. thermotolerans* Kr1 used for the subsequent genome sequencing was grown in the medium of the abovementioned composition, supplemented with 400 mM Zn^2+^. To determine 2-oxoglutarate dehydrogenase activity, *S. thermotolerans* Kr1 was grown under mixotrophic conditions at optimal (40°С) and nonoptimal (12 and 55°С) temperatures. The cultivation was carried out with mixing by air agitation (sterile air was supplied at a flow rate of 2 l min^–1^) in Redline RI53 incubators (Binder, Germany).

For measurements of antioxidant enzyme activities, *S. thermotolerans* Kr1 was cultivated in the modified 9K medium containing ferrous iron and yeast extract at atmospheric oxygen partial pressure or intense aeration. Methylcitrate synthase was determined in the extracts of the cells grown at atmospheric oxygen partial pressure in the medium containing ferrous iron, yeast extract, and propionate (0.02, 0.04, 0.06, and 0.08%).

### Microscopy and quantitative assessment of cells

Quantitative assessment of the strain Kr1 cells was carried out by direct counts in the Goryaev chamber and by the method of serial terminal tenfold dilutions^31^. A Mikmed-2 microscope (LOMO, Russia) equipped with a phase contrast device was used.

### Genome sequencing and assembly

Late-exponential cells of *S. thermotolerans* Kr1 were collected by centrifugation (10 000 *g*, 15 min, 4 °C) and washed twice with acidified 9K medium without energy sources (pH 1.8) for the subsequent genome sequencing. Total DNA was extracted by phenol/chloroform method with the lysis buffer containing the following: 1 M sucrose; 150 mM NaCl; 50 mM Tris HCl (pH 8.0); 50 mM EDTA; lysozyme, 2 mg/ml (Sigma, Germany); and proteinase K, 0.5 mg/ml (Sigma, Germany). Genome sequencing was performed using the Roche 454 Life Sciences Genome Sequencer GS FLX+ Genetic Analyzer (Roche 454 Life Science, United States) according to the standard protocol for a shotgun genome library. Assembly of raw sequencing reads with an average length of 694 bases was performed by the GS *de novo* assembly software Newbler version 2.9 (Roche 454 Life Science, United States). Primary assembly resulted in 46 contigs (>2000 bp in size) and an estimated genome size of 3.3 Mb. Relevant amplicons were generated and sequenced by conventional Sanger capillary methods on ABI Prism 3730 Genetic Analyzer (Applied Biosystems, United States; Hitachi, Japan) to fill the gaps between contigs. After gaps between contigs were filled, the size of the full sequence of the circular chromosome was 3 317 203 bp.

### Genome annotation and analysis

The complete genome sequence of *S. thermotolerans* Kr1 was annotated using Prokka v1.10^92^ and the NCBI Prokaryotic Genome Annotation Pipeline (United States, http://www.ncbi.nlm.nih.gov/genome/annotation_prok/).

Functional characterization of the genome of *S. thermotolerans* Kr1 was carried out using the KOALA (KEGG Orthology and Links Annotation) system^93^. Local BLAST (BlastX threshold e-value = 1 × 10^−6^, BLOSUM-62 matrix) and the nr database were used. Blast2GO was also used to analyze gene ontology and to functionally annotate genes (COG, PFAM, and signal peptides). G + C content and skew were calculated by gcSkew.pl script (https://github.com/abremges/2015-pseudo). Positions of the genes and plasmid insertions, COG, GC-skew, and G + C content were drawn using Circos^94^. MetaCyc^95^ and Pathway collages^96^ were used to construct metabolic maps.

Glimmer 3, a system for finding genes in microbial DNA, was used to analyze target regions containing the genes of interest^97,98^. Unknown proteins were annotated using the HHpred server for protein remote homology detection (https://toolkit.tuebingen.mpg.de/#/tools/hhpred). The annotation was manually improved by further comparison to the *Sulfobacillus* genomes. We used annotated genome sequences of 16 phylogenetically diverse strains assigned to *S. acidophilus*, *S. thermosulfidooxidans*, *S. benefaciens*, and *Sulfobacillus* spp. hq2 and UBA10397, available in the NCBI GenBank databases (https://www.ncbi.nlm.nih.gov/genome/?term=Sulfobacillus), for the nucleotide and protein sequence comparisons. The sequences were compared using the NCBI Basic Local Alignment Tool (United States, https://blast.ncbi.nlm.nih.gov/Blast.cgi) and by analysis of orthology groups (OG).

Protein sequences for OG were obtained from 16 *Sulfobacillus* strains using the NCBI databases. OGs were obtained using the OrthoFinder software with default parameters^99^. The phylogenetic tree showing the position of *Sulfobacillus* strains was constructed using the STAG (Species Tree Inference from All Genes) method (https://github.com/davidemms/STAG), which is built into the algorithms of the OrthoFinder software program^99^. The phylogenetic tree was visualized using FigTree v.1.4.3 (http://tree.bio.ed.ac.uk/software/figtree/).

### Plasmid-like region analysis

We used the method of GC profiling to identify the boundaries of the plasmid region in the chromosome^100,101^. The ICEberg web-based resource^102^ (http://db-mml.sjtu.edu.cn/ICEberg/) and the web-based ICEfinder tool for the detection of ICEs/IMEs in the bacterial genomes (http://202.120.12.136:7913/ICEfinder/ICEfinder.html) were also used to identify the integrated DNA elements. The candidate plasmid region containing the putative ICE was aligned with the pL15 plasmid sequence of *S. thermotolerans* L15 (NC_025041) and pY0017 sequence of *S. thermotolerans* Y0017 (NC_016040), using Mauve 2.4.0^103^ and BlastN algorithm (https://blast.ncbi.nlm.nih.gov/Blast.cgi). Visualization was carried out with the GenoPlotR package^104^.

### Enzyme assays

In order to determine the specific enzyme activities of 2-oxoglutarate dehydrogenase (OGDH, EC 1.2.4.2), superoxide dismutase (SOD, EC 1.15.1.1), glutathione (GSH) peroxidase (EC 1.11.1.9), and 2-methylcitrate synthase (MCS) (EC 2.3.3.5), late-exponential cells were collected by centrifugation (10 000 *g*, 15 min, 4 °C). The cell precipitates were washed twice with acidified 9K medium (pH 1.8) without energy sources and resuspended in 0.1 M Tris HCl buffer (pH 7.4) (OGDH), 0.05 M potassium phosphate buffer (pH 7.0) (SOD and GSH peroxidase), or HEPES-NaOH buffer (pH 7.1) (MCS). Cell-free extracts were obtained by pellet sonification using the UZDN-2T ultrasonic disintegrator (Russia) (1.5 min with 2-min intervals for cooling, four times; 22 Hz, 40 mA) and centrifuged at 40 000 *g* (4 °C, 30 min). The supernatant was assayed for the enzymatic activity of OGDH (nmol/min mg protein) judged from NAD reduction in the presence of 2-oxoglutarate, thiamine pyrophosphate, and coenzyme A^105^. SOD activity was determined in the cell-free extracts by the inhibition of the reduction of nitro blue tetrazolium (NBT); one unit of SOD activity was that amount of SOD, which inhibited the reaction by 50%^106^. GSH peroxidase activity was assayed in the cell-free extracts by a decrease in NADPH content in the reaction mixture^107^. MCS activity was determined by the oxalate-stimulating production of free propionyl-CoA^71^. A PE-5400UV spectrophotometer (ECROS, Russia) was used for enzyme activity assays. Protein content was measured by the Lowry method^108^.

### Alignments and phylogenetic analysis of proteins

The protein sequences were aligned using the NCBI BlastP algorithm (https://blast.ncbi.nlm.nih.gov/Blast.cgi), MEGA7^109^, and the Lasergene software package ver. 8.1.3(4) (DNASTAR, Inc., United States).

The phylogenetic tree was constructed using the MEGA7^109^ software package. Evolutionary history was inferred using the neighbor-joining method^110^ and amino acid sequences of phylogenetically close microbial proteins. The bootstrap test (1000 replicates) was applied^111^. The evolutionary distances were computed using the Poisson correction method^112^ and are in the units of the number of amino acid substitutions per site.

### Statistics

The experiments, including enzyme assays, were carried out in three parallels with 3–5 replicates; the significance of the results was assessed using the Student’s t-test at the significance level P ≤ 0.05.

## Supplementary information

Supplementary Information

## Data Availability

The genome sequence of *S. thermotolerans* Kr1 was deposited in the GenBank databases under the accession number CP019454 and is public at https://www.ncbi.nlm.nih.gov/nuccore/CP019454.1?report=genbank. Sequence read achieve (SRA) was submitted under SRA accession number SRP143515.
