# Supplementary material for: Sulfobacillus thermotolerans: new insights into resistance and metabolic capacities of acidophilic chemolithotrophs
Source: Sci Rep. 2019 Oct 21;9:15069. doi: 10.1038/s41598-019-51486-1 (PMC6803676; doi:10.1038/s41598-019-51486-1)
Supplement: Supplementary file 1 — Supplementary Information [file 41598_2019_51486_MOESM1_ESM.pdf]

Sulfobacillus thermotolerans: new insights into resistance and metabolic capacities of acidophilic chemolithotrophs

Anna E. Panyushkina, Vladislav V. Babenko, Anastasia S. Nikitina, Oksana V. Selezneva, Iraida A. Tsaplina, Maria A. Letarova, Elena S. Kostryukova, and Andrey V. Letarov

SUPPLEMENTARY INFORMATION LEGENDS

Figure S1. GC profile of the plasmid-like region of *Sulfobacillus thermotolerans* Kr1.

Figure S2. Alignment of the nucleotide sequence of the plasmid-like region of *Sulfobacillus thermotolerans* Kr1 with the plasmid sequences of *S. thermotolerans* Y0017 (a) and *S. thermotolerans* L15 (b). Mauve algorithm was used for the alignment of the sequences (MAUVE version 2.4.0), where regions of the same color indicate high similarity and are connected by the bars of the same color.

Figure S3. Alignment of the amino acid sequences of two rusticyanin proteins encoded in the genome of *S. thermotolerans* Kr1. The Lipman-Pearson alignment was performed using the MegAlign tool of the Lasergene software package ver. 8.1.3(4) (DNASTAR, United States).

Table S1. The unique genes and proteins, identified in the genome of *S. thermotolerans* Kr1. A comparison to other strains of the genus *Sulfobacillus* was carried out by analysis of orthology groups (OG). Protein sequences for OG were obtained from 16 *Sulfobacillus* strains using the NCBI databases. OGs were obtained using the OrthoFinder software with default parameters[99]. Putative homologs were identified using the NCBI BlastP algorithm (https://blast.ncbi.nlm.nih.gov/Blast.cgi). Protein remote homology was detected using the HHpred server (https://toolkit.tuebingen.mpg.de/#/tools/hhpred).

Table S2. Characterization of the chromosome integrated plasmid of *Sulfobacillus thermotolerans* Kr1.

Table S3. Enzymes of the TCA cycle and glyoxylate cycle encoded in the genome of *Sulfobacillus thermotolerans* Kr1.

Table S4. Enzymes of the methylcitrate cycle encoded in the genome of *Sulfobacillus thermotolerans* Kr1.

Table S5. Proteins of oxalate degradation pathway encoded in the genome of *Sulfobacillus thermotolerans* Kr1.

Table S6. Electron transfer components encoded in the genome of *Sulfobacillus thermotolerans* Kr1.

Table S7. Proteins predicted to participate in stress resistance and defense systems in *Sulfobacillus thermotolerans* Kr1.

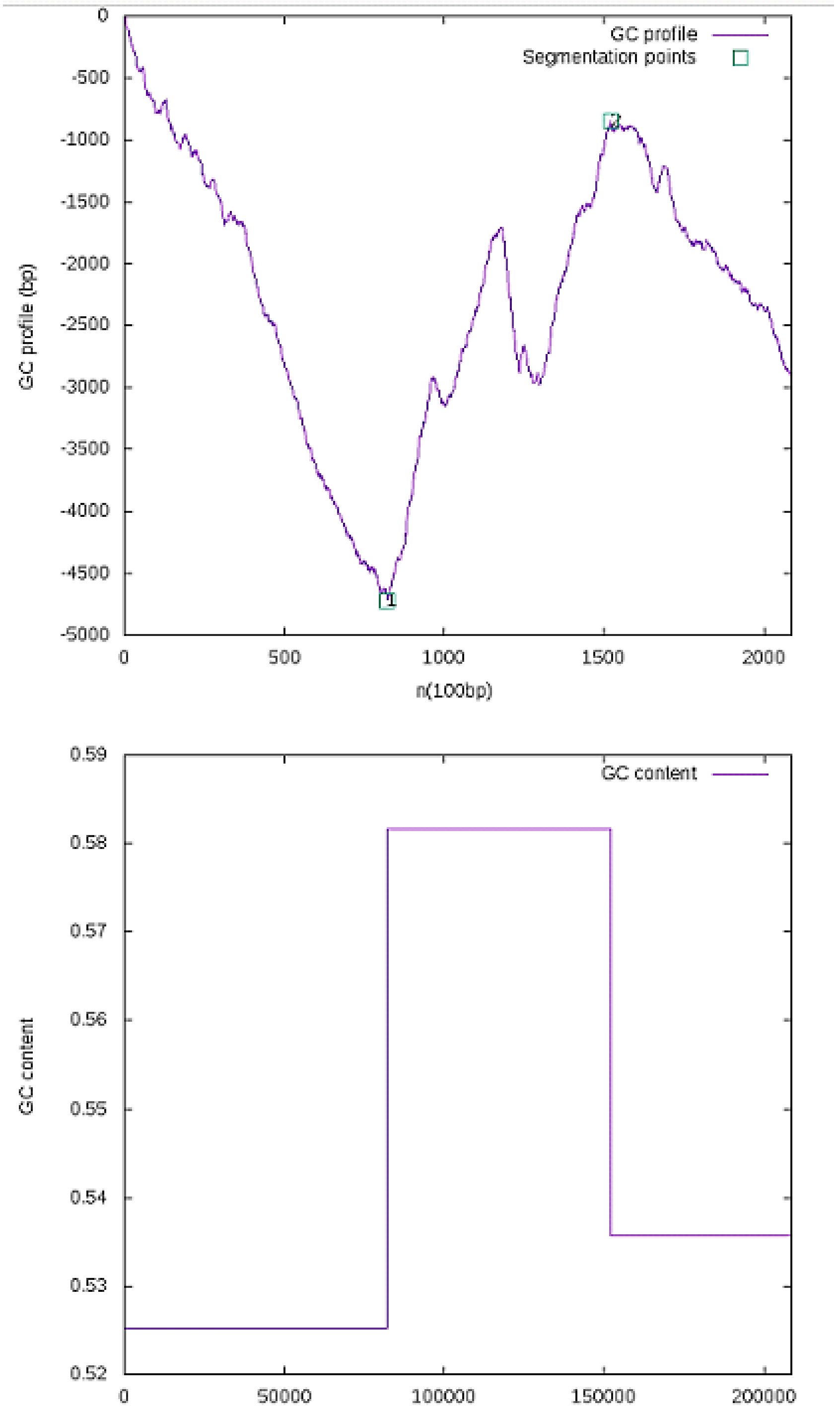

Figure S1. GC profile of the plasmid-like region of *Sulfobacillus thermotolerans* Kr1.

a

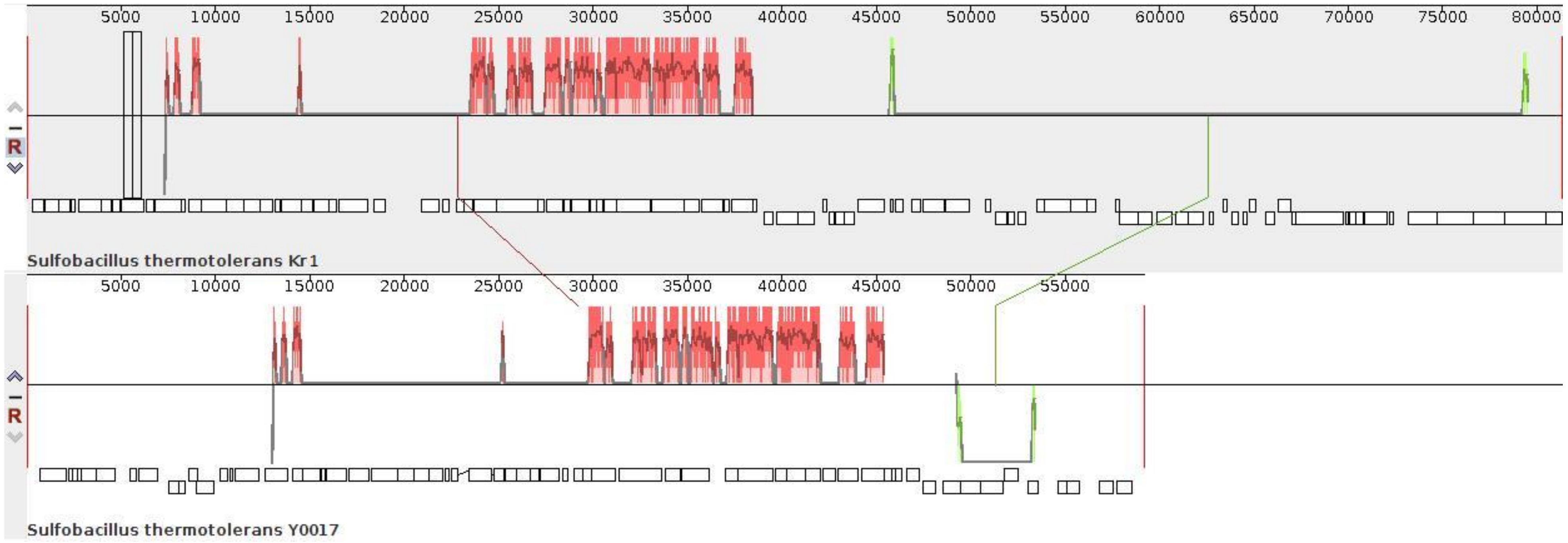
5000 10000 15000 20000 25000 30000 35000 40000 45000 50000 55000 60000 65000 70000 75000 80000
R
Sulfobacillus thermotolerans Kr1
5000 10000 15000 20000 25000 30000 35000 40000 45000 50000 55000
R
Sulfobacillus thermotolerans Y0017

b

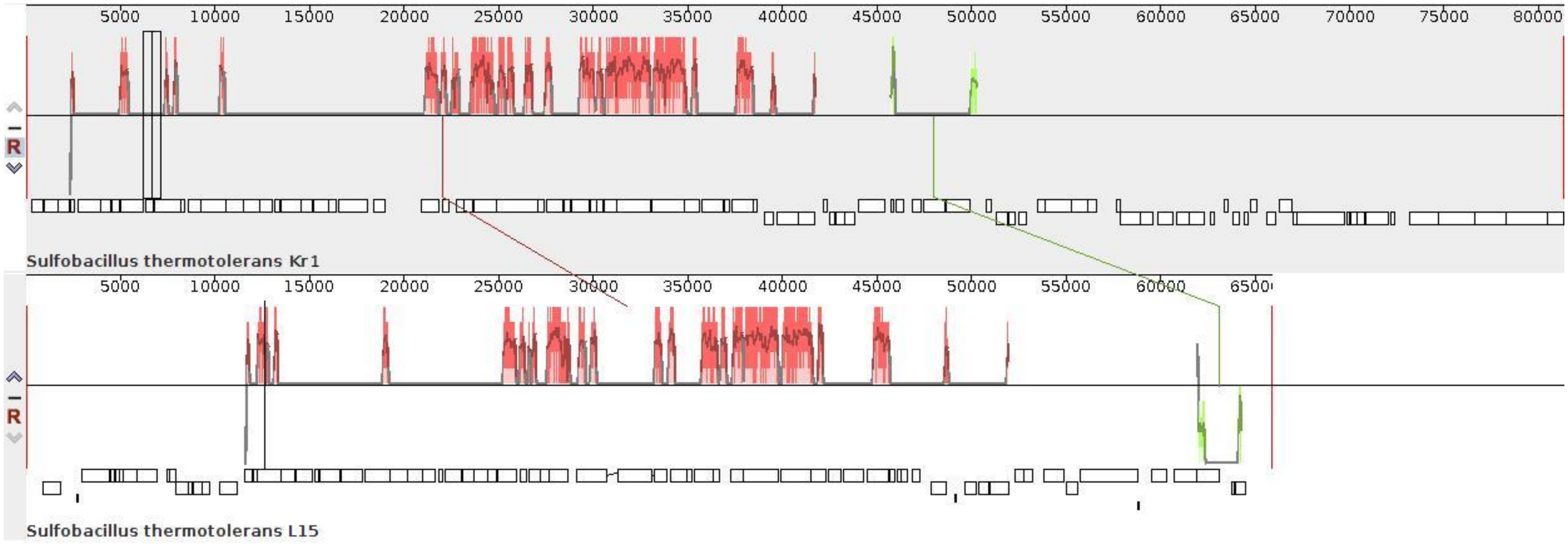

Figure S2. Alignment of the nucleotide sequence of the plasmid-like region of *Sulfobacillus thermotolerans* Kr1 with the plasmid sequences of *S. thermotolerans* Y0017 (a) and *S. thermotolerans* L15 (b)**.** Mauve algorithm was used for the alignment of the sequences (MAUVE version 2.4.0), where regions of the same color indicate high similarity and are connected by the bars of the same color.

```
Lipman-Pearson Protein Alignment
Ktuple: 2; Gap Penalty: 4; Gap Length Penalty: 12
Seq1(1>258)                   Seq2(1>161)                Similarity   Gap     Gap  Consensus
BXT84_00770 rusticyanin.pro   BXT84_05090 rusticyanin.pro     Index  Number  Length     Length
(103>258)                     (9>158)                          29.6       5       8        157

                                 v100      v110      v120      v130      v140
BXT84_00770 rusticyanin.pro GAASGSVWTPAQVAKLVQQSEQGVTIDRTTNTITYHSTQALLVPLAAPAA
                             ..     T::: :.:.  : .:.::D. .N I.:H: Q : : :AA : 
BXT84_05090 rusticyanin.pro MDQHLQSATSTHFTHFAMTPPPDASVDSQENRIIFHN-QHVQMDVAALSR
                                     ^10       ^20       ^30       ^40
                                 v150      v160      v170      v180      v190
BXT84_00770 rusticyanin.pro LHIKGMQWEIDGLINPKVVVPQGAQITVDLVNADQGYLHGFDVTTARPPF
                             ::.:: .:I:GL :P:::VP G:::TV:: N.D :  HG. :.:.:PPF
BXT84_05090 rusticyanin.pro -EFSDVMFHIGGLDDPRIIVPLGSDVTVNFQNQDTANPHGWRLIADKPPF
                             ^50       ^60       ^70       ^80       ^90
                                  v200      v210      v220      v230      v240
BXT84_00770 rusticyanin.pro RE-MAMMQGPAAFSGAFIMPILPETSQGQYHRSTQFTATTAGTYYYICPV
                            ::  :  : P AF G: :  . P:.S GQ H    FTA. AG.Y YICPV
BXT84_05090 rusticyanin.pro QNPQSAAHKPLAFVGSEVGVTNPQES-GQAH----FTANQAGIYTYICPV
                             ^100      ^110      ^120           ^130      ^140
                                  v250
BXT84_00770 rusticyanin.pro PGHAAQGMAGQFVVA
                            :.::: G: G F V:
BXT84_05090 rusticyanin.pro SEDGGIGLYGIFEVS
                                  ^150
```

Figure S3. Alignment of the amino acid sequences of two rusticyanin proteins encoded in the genome of *S. thermotolerans* Kr1. The Lipman-Pearson alignment was performed using the MegAlign tool of the Lasergene software package ver. 8.1.3(4) (DNASTAR, United States).

Table S1. The unique genes and proteins, identified in the genome of *S. thermotolerans* Kr1. A comparison to other strains of the genus *Sulfobacillus* was carried out by analysis of orthology groups (OG). Protein sequences for OG were obtained from 16 *Sulfobacillus* strains using the NCBI databases. OGs were obtained using the OrthoFinder software with default parameters[99]. Putative homologs were identified using the NCBI BlastP algorithm (https://blast.ncbi.nlm.nih.gov/Blast.cgi). Protein remote homology was detected using the HHpred server (https://toolkit.tuebingen.mpg.de/#/tools/hhpred).

No.	CDS (Protein ID)	Position	Homolog(s) (NCBI annotation)	Putative protein (function) (BlastP)/ remote protein homolog (HHpred)
1	BXT84_00165	37919–38122	Hypothetical protein	3C-like protease
2	BXT84_00175	39905–40084	Hypothetical protein	Oxidative stress-induced growth inhibitor 2
3	BXT84_00455	83753–84163	Hypothetical protein	Prephenate dehydrogenase
4	BXT84_00460	84206–85441	Hypothetical protein	MinD-like protein
5	BXT84_00465	85578–85949	Hypothetical protein	Nuclear pore complex protein NUP155
6	BXT84_00570	106291–106647	Hypothetical protein	WYL domain-containing DNA-binding transcriptional regulator
7	BXT84_00580	107687–107977	Hypothetical protein	Putative conjugation protein TrsB
8	BXT84_00590	109060–109377	Hypothetical protein	DNA-directed RNA polymerase (EC:2.7.7.6)
9	BXT84_00595	109377–109712	Hypothetical protein	Membrane protein
10	BXT84_00605	110429–112216	Hypothetical protein	VirB4-like type IV secretory system protein
11	BXT84_00625	116127–116402	Hypothetical protein	mRNA interferase YafQ (E.C.3.1.-.-)
12	BXT84_00635	117675–117893	Hypothetical protein	Amidase (E.C.3.5.1.4)
13	BXT84_00645	118941–120038	Hypothetical protein	EcoT38I restriction endonuclease
14	BXT84_00655	121391–121570	Hypothetical protein	Omega transcriptional repressor
15	BXT84_00660	121699–121980	Hypothetical protein	Agenet domain-containing protein
16	BXT84_00665	122045–122533	Hypothetical protein	DUF488 domain-containing protein
17	BXT84_00670	122520–123071	Hypothetical protein	Protein containing DUF1130
18	BXT84_00685	125238–125606	Hypothetical protein	Ribonuclease VapC11 (E.C.3.1.-.-), Antitoxin VapB11
19	BXT84_00875	162211–162579	Hypothetical protein	Viral protein
20	BXT84_00880	162608–162820	Hypothetical protein	Cytochrome b_6-f complex
21	BXT84_00885	162839–163636	Hypothetical protein	Immunoglobulin G-binding protein A

22	BXT84_00925	167048–167353	Hypothetical protein	Transcriptional regulator
23	BXT84_02040	387531–388112	Hypothetical protein	Proprotein convertase subtilisin
24	BXT84_03020	564497–564700	Hypothetical protein	N-acetylgalactosaminyl transferase (o-glycan biosynthesis)
25	BXT84_03025	565511–566686	Hypothetical protein	Uncharacterized protein
26	BXT84_03030	566683–567192	Hypothetical protein	Signal transducer and activator
27	BXT84_03035	567189–569387	Hypothetical protein	AYP/GTP-binding protein
28	BXT84_03045	571290–572168	TIGR02391 family protein	Associated with phage and plasmid regions, restriction system loci, transposons, and housekeeping genes (function unknown)
29	BXT84_03065	577807–579894	Hypothetical protein	DUF262 domain-containing protein;<br>Uncharacterized conserved protein, contains ParB-like and HNH nuclease domains (function unknown)
30	BXT84_03075	582562–582744	Hypothetical protein	Zinc Metalloprotease ZMP1 (E.C.3.4.24.-)
31	BXT84_03150	595958–596209	Hypothetical protein	Mobile element protein; transposase proteins necessary for efficient DNA transposition
32	BXT84_03160	597302–601213	Restriction endonuclease	Restriction
33	BXT84_04170	814432–814872	Hypothetical protein	Cell density-dependent motility repressor / LysR transcriptional regulator
34	BXT84_04850	944887–945090	Hypothetical protein	Protein THO1
35	BXT84_07475	1454760–1454948	Hypothetical protein	Prion-like protein
36	BXT84_07965	1555353–1555652	Hypothetical protein	Flavodoxin; electron transfer, cytoplasma
37	BXT84_07975	1556167–1556358	Hypothetical protein	Protein (ANNEXIN I); domain 1, metal transport
38	BXT84_08105	1587905–1588345	Hypothetical protein	E3 ubiquitin-protein ligase HECTD1 (E.C.6.3.2.-)
39	BXT84_09230	1827958–1828305	Hypothetical protein	Mono-ADP-ribosyl transferase C3 (E.C.2.4.2.-)
40	BXT84_10130	2018881–2019147	Hypothetical protein	OProtein HP0242
41	BXT84_10485	2094784–2096991	Hypothetical protein	Alpha-galactosidase; hydrolysis of α-1,6-linked α-galactose residues from oligosaccharides (melibiose, raffinose, and stachyose)
42	BXT84_10490	2096997–2099012	Beta-galactosidase	Breaks down the disaccharide lactose into glucose and galactose
43	BXT84_10495	2099005–2100033	Hypothetical protein	Lac I family transcriptional regulator; regulates carbohydrate utilization genes
44	BXT84_10500	2100074–2100901	Hypothetical protein	Carbohydrate ABC transporter permease
45	BXT84_10505	2100898–2101746	Lactose ABC transporter permease	Transport of lactose
46	BXT84_10510	2101878–2103191	Hypothetical protein	Sugar ABC transporter substrate-binding protein
47	BXT84_10540	2110709–2112091	Hypothetical protein	Putative glycosyltransferase
48	BXT84_10585	2121132–2121329	Hypothetical protein	MFS transporter
49	BXT84_10985	2220567–2221598	Hypothetical protein	Natterin-like protein, aerolysin-like toxin; may bind mono- or oligosaccharides with

				high specificity
50	BXT84_11205	2270260–2270496	Hypothetical protein	Clostripain-related protein, Peptide Inhibitor BTN-VLTK-AOMK
51	BXT84_12370	2530468–2530734	Hypothetical protein	CREB-binding protein (E.C.2.3.1.48)
52	BXT84_12625	2578571–2578789	Hypothetical protein	Site-specific integrase/recombinase XerD related protein; integrase
53	BXT84_12630	2578991–2579281	Hypothetical protein	Methyltransferase domain protein
54	BXT84_12650	2581195–2581494	Hypothetical protein	MFS transporter
55	BXT84_12655	2581545–2581778	Hypothetical protein	TetR family transcriptional regulator
56	BXT84_12670	2583453–2583836	Hypothetical protein	mRNA interferase RelE (E.C.3.1.-.-)
57	BXT84_12685	2585485–2585688	Hypothetical protein	DUF2029 domain-containing protein
58	BXT84_12690	2585952–2586323	Hypothetical protein	Secreted metalloprotease Mcp02
59	BXT84_12700	2588450–2589694	Hypothetical protein	Subunit alpha, Subunit beta; Farnesyl transferase, transferase
60	BXT84_12825	2610809–2611180	Hypothetical protein	ATP-dependent DNA helicase
61	BXT84_12835	2612984–2613184	Hypothetical protein	Alpha trypsin
62	BXT84_14040	2853620–2853799	Hypothetical protein	Viral protein
63	BXT84_14045	2853942–2855810	Hypothetical protein	Phage- or plasmid-associated DNA primase
64	BXT84_14050	2855889–2856266	Hypothetical protein	GITR ligand; TNF, GITR
65	BXT84_14055	2856634–2856843	Hypothetical protein	Tetratricopeptide repeat protein
66	BXT84_14060	2857015–2857356	Hypothetical protein	Putative rRNA methyltransferase (E.C.2.1.1.179)
67	BXT84_14070	2860439–2861035	Hypothetical protein	Z-DNA-binding protein
68	BXT84_14080	2863093–2863611	Hypothetical protein	UDP-N-acetylmuramoyl-tripeptide-D-alanyl-D-alanine ligase; synthesis of bacterial wall peptidoglycan
69	BXT84_14085	2863663–2864064	Hypothetical protein	L-D-alanine ligase (E.C.6.3.2.10)
70	BXT84_14090	2864148–2864351	Hypothetical protein	Fimbrial protein; pilus subunit, extracellular, cell adhesion
71	BXT84_14095	2864390–2865229	Hypothetical protein	OmpA family protein / Epidermal growth factor receptor (E.C.2.7.10.1)
72	BXT84_14105	2866283–2866546	Hypothetical protein	Dimer, bacterial conjugation, relaxase, DNA
73	BXT84_14605	2949032–2949283	Hypothetical protein	Potassium voltage-gated channel subfamily E
74	BXT84_14750	2977225–2977503	Hypothetical protein	Envelope glycoprotein B
75	BXT84_15330	3107946–3108137	Hypothetical protein	Avirulence protein; Ptha, 1.5 repeat units;
76	BXT84_16020	3265260–3266084	Hypothetical protein	Lactose permease; transport
77	BXT84_16035	3268041–3268502	Hypothetical protein	Bicyclomycin resistance protein TcaB; alpha helical transmembrane protein
78	BXT84_16040	3268755–3269099	Hypothetical protein	TraF protein, TraO protein, TraN protein / c-type cytochrome
79	BXT84_16045	3269209–3269472	Hypothetical protein	Yop proteins translocation protein U; autocleavage, Type III secretion system
80	BXT84_16055	3270656–3270946	Hypothetical protein	DNA-binding protein, SSDNA-binding protein

Table S2. Characterization of the chromosome integrated plasmid of *Sulfobacillus thermotolerans* Kr1

CDS (Protein ID)	Position	Protein size, a. a.	Putative homolog(s)	Function (reference(s))	The closest relative protein, description; % aa identity (% aa coverage), E-value; accession number	pL15 and pY0017 homolog; % aa identity (% aa coverage), E-value	
						pL15	pY0017
BXT84_00415	77249-78193	314	Hypothetical protein	Metallo-beta-lactamase superfamily	MBL fold metallo-hydrolase; *Sulfobacillus thermosulfidooxidans* ; 97.1% (100%), 0; PSR37807.1	n.d.[c]	n.d.
BXT84_00420	78339-79025	228	Hypothetical protein	Serine protease (EC 3.4.21.-)(Posttranslational modification, protein turnover, chaperones)	PDZ-domain containing protein *Sulfobacillus* sp.; 99.5% (96%); 1e-153; WP_103374024.1	n.d.	n.d.
BXT84_00425	79534-80112	192	Hypothetical protein	-	Hypothetical protein; *Treponema succinifaciens*; 46% (19%), 1.1; WP_013700615	n.d.	n.d.
BXT84_00430	80157-80906	249	Hypothetical protein	-	Hypothetical protein; *S. thermosulfidooxidans*; 52% (99%), 9e-88; WP_020373029	n.d.	n.d.
BXT84_00435	80897-81529	210	Hypothetical protein		No significant similarity found		
BXT84_00440	81569-81778	69	Hypothetical protein	-	Helix-turn-helix domain-containing protein; *Desulfotomaculum geothermicum*; 45% (81%), 3e-07;_SFR15022	n.d.	n.d.
BXT84_00445	81984-83180	398	Hypothetical protein	-	Helix-turn-helix domain-containing protein; *Desulfotomaculum geothermicum*; 40% (44%), 8e-38; SFR15022	n.d.	n.d.
BXT84	83177-83683	168	Hypothetical	-	Hypothetical protein; *Mahella*	n.d.	n.d.

_00450			protein		*australiensis*; 33% (98%), 5e-19; WP_021168117		
BXT84 _00455	83753-84163	136	Hypothetical protein		No significant similarity found		
BXT84 _00460	84206-85441	411	MinD-like protein[a]	Cell division ATPase, a member of the MinD/ParA superfamily of ATPases[1, d]	MinD-like protein, ***S. thermotolerans*** pY0017; 36% (51%), 3e-24; WP_014106994	MinD-like protein; 35% (51%), 3e-23	MinD-like protein; 36% (51%), 3e-24
BXT84 _00465	85578-85949	123	Hypothetical protein	-	Hypothetical protein, *Paenibacillus* sp. Soil766; 39% (43%), 0.28; WP_057314864	-	-
BXT84 _00470	86015-87388	457	MinD	Cell division ATPase, a member of the MinD/ParA superfamily of ATPases[1]	Hypothetical protein, *S. thermosulfidooxidans;* 61% (98%), 2e-176; WP_053958145	MinD; 42% (60%), 9e-53	MinD; 43% (60%), 6e-53
BXT84 _00475	87385-87615	76	Vitamin K epoxide reductase	Reduction of vitamin K 2,3-epoxide and vitamin K to vitamin K hydroquinone[2]	Hypothetical protein, ***S. thermotolerans*** pL15; 56% (76%), 4e-08; WP_031942634	Hypothetical protein; 56% (76%), 4e-08	Hypothetical protein; 52% (71%), 5e-07
-	87640-87768	42	Hypothetical protein (Identified by Glimmer 3)	-	Hypothetical protein, ***S. thermotolerans*** pL15; 73% (71%), 8e-06; WP_031942634	Hypothetical protein; 73% (71%), 8e-06	Hypothetical protein; 70% (71%), 2e-05
BXT84 _00480	87771-88472	233	Hypothetical protein	-	Hypothetical protein, *S. thermosulfidooxidans;* 65% (99%), 2e-100; WP_053958144	Hypothetical protein; 52% (84%), 4e-58	Hypothetical protein 62% (67%), 1e-58
BXT84 _00485	88490-89815	441	CpaF\ TadA	Flp pilus assembly ATPase-like protein. Provide the energy to power the DNA transport process[1]	CpaF-like hypothetical protein, *S. thermosulfidooxidans*; 62% (94%), 1e-172; WP_053958143	CpaF\TadA; 46% (87%), 8e-95	CpaF\TadA; 45% (93%), 2e-97
BXT84 _00490	89820-90692	290	TadB	Putative type II secretion system/ pilus assembly protein. TadB and TadC are putative integral inner membrane proteins, forming a heteromultimer that allows the passage of the other Tad components across the inner membrane[1]	Hypothetical protein, *S. thermosulfidooxidans*; 54% (98%), 6e-98; WP_053958142	TadB-like protein; 38% (98%), 4e-47	TadB-like protein; 38% (98%), 3e-46
BXT84 _00495	90722-91549	275	TadC		Hypothetical protein, *S. thermosulfidooxidans*; 57% (99%), 2e-103; WP_053958143	TadC-like protein; 37% (93%), 9e-36	TadC-like protein; 37% (93%), 1e-34

BXT84_00500	91563-92225	220	Hypothetical protein	-	Hypothetical protein, *S. thermosulfidooxidans*; 62% (94%), 4e-79; WP_053958140	Hypothetical protein; 37% (70%), 5e-15	Hypothetical protein; 37% (70%), 3e-14
BXT84_00505	92352-92615	87	Hypothetical protein	-	Hypothetical protein, *S. thermosulfidooxidans*; 65% (52%), 9e-09; WP_053958139	n.d.	n.d.
BXT84_00510	92685-93743	352	Hypothetical protein	-	Hypothetical protein, *S. thermosulfidooxidans*; 55% (97%), 5e-113; WP_053958138	Hypothetical protein; 35% (99%), 2e-26	Hypothetical protein; 35% (99%), 6e-24
BXT84_00515	93734-94354	206	Hypothetical protein	-	Hypothetical protein, *S. thermosulfidooxidans*; 60% (90%), 4e-63; WP_053958137	n.d.	n.d.
BXT84_00520	94452-95201	249	Hypothetical protein	-	Hypothetical protein, *S. thermosulfidooxidans*; 61% (88%), 6e-84; WP_053958136	n.d.	n.d.
BXT84_00525	95198-95638	146	Hypothetical protein	-	Hypothetical protein, *S. thermosulfidooxidans*; 52% (93%), 4e-26; WP_053958135	n.d.	n.d.
BXT84_00530	95728-97248	506	Hypothetical protein	-	Hypothetical protein, *S. thermosulfidooxidans*; 64% (95%), 0.0; WP_053958134	n.d.	n.d.
BXT84_00535	97607-98194	195	Hypothetical protein	-	Hypothetical protein, *S. thermosulfidooxidans*; 55% (97%), 2e-69; WP_053958133	n.d.	n.d.
BXT84_00540	100094-101047	317	StbA	Plasmid segregation stability/ partitioning protein ParM/ StbA, which may serve as a stability locus[3]	StbA-family protein, ***S. thermotolerans*** pY0017; 46% (99%), 8e-78; WP_014107010	StbA-like protein; 46% (99%), 3e-77	StbA-like protein; 46% (99%), 8e-78
BXT84_00545	101245-101556	103	Hypothetical protein	-	Hypothetical protein, ***S. thermotolerans*** pY0017; 39% (82%), 2e-07; WP_014107011	Hypothetical protein; 38% (82%), 7e-07	Hypothetical protein; 39% (82%), 2e-07
BXT84_00550	101958-102425	155	MobC-like mobilization protein	Belongs to the group of relaxases; binds to a single cis-active site of a mobilizing	Hypothetical protein, ***S. thermotolerans*** pL15; 41% (49%), 2e-05; WP_031942649	n.d.	Hypothetical protein, 41% (49%), 2e-05

				plasmid, oriT region[4]			
BXT84 _00555	102409-102870	153	Hypothetical protein	CopG transcriptional regulator involved in the control of plasmid copy number	CopG family transcriptional regulator; *Deltaproteobacteria bacterium* RBG_13_47_9; 33% (23%), 5.6; OGP64621	n.d.	n.d.
BXT84 _00560	102900-104105	401	Relaxase	MOB$_P$-type family relaxase is essential for conjugative plasmids, initiates and terminates conjugative DNA processing[5]	MOBP-family relaxase, ***S. thermotolerans*** pLY0017; 49% (100%), 5e-111; WP_014107014	Relaxase, 52% (81%), 3e-99	Relaxase, 49% (100%), 5e-111
BXT84 _00565	104111-106258	715	TraG	ATPase-like protein involved in conjugal plasmid transfer. TraG-like type IV coupling proteins are VirD4 homologs required to link the DNA-protein (relaxosome) substrate to the transmembrane transfer complex[1,6]	TraG, ***S. thermotolerans*** pL15; 39% (98%), 2e-148; WP_031942652	TraG; 39% (98%), 2e-148	TraG; 38% (98%), 3e-148
BXT84 _00570	106291-106647	118	Hypothetical protein, hydrolase	Predicted DNA-binding transcriptional regulator YafY, contains HTH and WYL domains	Bacterium OL-1; 41% (37%), 3.1; WP_032110088; hydrolase TatD; *Ruminobacter* sp. RM87; 44% (36%), 4.4	n.d.	n.d.
BXT84 _00575	106717-107607	296	LtrC	Primase, similar to replication primases	LtrC-like protein; ***S. thermotolerans*** pL15; 52% (88%), 2e-83; WP_031942654	LtrC-like protein; 52% (88%), 2e-83	LtrC-like protein; 53% (83%), 2e-79
BXT84 _00580	107687-107977	96	TrsB	Putative conjugation protein similar to TrsB/ TrsL-like transfer complex protein of *Bacillus thuringiensis* and proteins TrsB/TraB and TrsL/TraL of *Staphylococcus aureus* conjugative plasmid pG01[1,7]	TrsB; ***S. thermotolerans*** L15 plasmid L15; 72% (95%), 1e-36; WP_031942655	TrsB, 72% (95%), 1e-36	-
BXT84 _00585	108050-109033	327	TrsL		TrsL; ***S. thermotolerans*** L15 plasmid L15; 45% (97%), 2e-76; WP_031942656	TrsL, 45% (97%), 2e-76	TrsL, 47% (85%), 2e-62
BXT84 _00590	109060-109377	105	Hypothetical protein	-	Hypothetical protein; ***S. thermotolerans*** pL15; 45%	Hypothetical protein; 45%	n.d.

					(57%), 6e-06; WP_031942657	(57%), 6e-06	
BXT84 _00595	109377-109712	111	Hypothetical protein	-	Membrane protein; *Bacillus cereus* group; 33% (92%), 9e-08; WP_000390116	n.d.	n.d.
BXT84 _00600	109777-110436	219	Hypothetical protein	-	Hypothetical protein; ***S. thermotolerans*** pY0017; 59% (86%), 2e-75; WP_014107018	Hypothetical protein; 58% (86%), 2e-75	Hypothetical protein; 59% (86%), 2e-75
BXT84 _00605	110429-112216	595	VirB4	VirB4-like type IV secretory system protein[8,9]	VirB4-like protein; ***S. thermotolerans*** pY0017; 56% (98%), 0.0; WP_014107019	VirB4; 56% (97%), 0.0	VirB4; 56% (98%), 0.0
BXT84 _00610	112297-114018	573	Lytic transglycosylase	Soluble lytic murein transglycosylase. Responsible for creating space within the peptidoglycan sacculus for its biosynthesis and recycling, cell division, and the insertion of flagella and secretion systems[10]	Lytic transglycosylase; ***S. thermotolerans*** L15 plasmid L15; 58% (88%), 0.0; WP_031942660	Lytic transglyco-sylase; 58% (88%), 0.0	Lytic trans-glycosylase; 60% (54%), 2e-109
BXT84 _00615	114047-114844	265	Hypothetical protein	-	Hypothetical protein; ***S. thermotolerans*** Y0017 plasmid pY0017; 39% (99%), 6e-41; WP_014107022	Hypothetical protein; 39% (99%), 7e-41	Hypothetical protein; 39% (99%), 6e-41
BXT84 _00620	114952-116073	373	Hypothetical protein, Putative Toprim	Putative Toprim-like protein with topoisomerase-primase domain found in type IA, IIA and IIB topoisomerases, bacterial DnaG-type primases, small primase-like proteins from bacteria and archaea, OLD family nucleases from bacteria and archaea, and bacterial DNA repair proteins of the RecR/M family[11]	Hypothetical protein (putative Toprim); ***S. thermotolerans*** pL15; 42% (87%), 4e-64; WP_031942663	Hypothetical protein (putative Toprim); 42% (87%), 4e-64	Hypothetical protein (putative Toprim); 42% (87%), 2e-63
BXT84 _00625	116127-116402	91	Hypothetical protein		No significant similarity found		

BXT84_00630	116563-117693	376	Phosphatidyl-serine/ Phosphatidyl-glycero-phosphate/ cardiolipin synthase-like protein	Catalyzes the reversible phosphatidyl group transfer from one phosphatidylglycerol molecule to another to form cardiolipin (CL) (diphosphatidylglycerol) and glycerol (by similarity). Synthesis of acidic phospholipids, phosphatidylglycerol and cardiolipin, which play specific roles in various cellular processes and are believed to be essential for cell viability[12]	Phosphatidylserine/phosphate-dylglycerophosphate/cardiolipin synthase-like protein; ***S. thermotolerans*** pL15; 52% (89%), 2e-109; WP_031942664	Phosphati-dylserine/ Phosphati-dylglycero-phosphate/ cardiolipin synthase-like protein; 52% (89%), 2e-109	Phosphatidylserine/phosphatidylglycerophosphate/cardiolipin synthase-like protein; 50% (89%), 1e-104
BXT84_00635	117675-117893	72	Hypothetical protein	No significant similarity found			
BXT84_00640	Complement (118256-118738)	160	Hypothetical protein	-	Hypothetical protein; *S. thermosulfidooxidans*; 39% (78%), 6e-22; WP_020376272	n.d.	n.d.
BXT84_00645	Complement (118941-120038)	365	EcoT38I restriction endonuclease	Restrictase, cleaves DNA at recognition site	SacI restriction endonuclease; *Sphingopyxis* sp. Root1497; 34% (95%), 2e-53; WP_056345818	n.d.	n.d.
BXT84_00650	Complement (120057-120911)	284	Methyl-transferase	DNA methylation	DNA-metyltransferase; *Alicyclobacillus acidocaldarius*; 77% (92%), 5e-152; WP_012810536	n.d.	n.d.
BXT84_00655	121391-121570	59	Hypothetical protein	-	Hypothetical protein; *Paenibacillus popilliae*; 55% (49%), 0.025; WP_006285995	n.d.	n.d.
BXT84_00660	Complement (121699-121980)	93	Hypothetical protein	-	Hypothetical protein; *Bacillus* sp. FJAT-27225; 40% (98%), 6e-07; WP_066202247	n.d.	n.d.
BXT84_00665	Complement (122045-122533)	162	Hypothetical protein	-	Hypothetical protein; *Prevotella oulorum*; 44% (98%), 6e-43;	n.d.	n.d.

					WP_025070017		
BXT84_00670	Complement (122520-123071)	183	Hypothetical protein	-	Hypothetical protein; *Alicyclobacillus shizuokensis*; 57% (96%), 4e-69; WP_067929591	n.d.	n.d.
BXT84_00675	123274-124638	454	Transposase, ISNCY-family protein	DNA transposition. The catalytic activity of this enzyme involves DNA cleavage at a specific site followed by a strand transfer reaction[13]	ISNCY family transposase ISCde2; Candidatus *Desulforudis audaxviator*; 44% (99%), 8e-118; WP_012301814	n.d.	n.d.
-	125025-125150	138	Serine recombinase (Identified by Glimmer 3)	Site-specific DNA recombinase (resolvase/ invertase) related to the DNA invertase Pin (Replication, recombination and repair)	Hypothetical protein; *Polymorphum gilvum*; 72% (92%), 2e-10; WP_013651402	Invertase recombinase -like protein; 90% (73%), 8e-10	Resolvase-like protein; 90% (73%), 9e-10
BXT84_00685	125238-125606	122	Hypothetical protein	-	Hypothetical protein; *Frankia* sp. EAN1pec; 35% (78%), 7e-15; WP_020460343	n.d.	n.d.
BXT84_00690	126070-126528	152	Hypothetical protein	-	Hypothetical protein; *S. thermosulfidooxidans*; 86% (100%), 0.0; WP_053958068	n.d.	n.d.
BXT84_00695	126678-127832	384	Hypothetical protein	Contains XRE-family HTH domain	Transcriptional regulator; *S. thermosulfidooxidans*; 91% (100%), 0.0; WP_053958067	n.d.	n.d.
BXT84_00700	127907-129145	412	NgoFVII restriction endonuclease	Restrictase, cleaves DNA at recognition site	NgoFVII restriction endonuclease; *S. acidophilus* TPY; 56% (66%), 8e-95; AEJ38753	n.d.	n.d.
-	129374-129544	56	Cation transporter[b] (Identified by Glimmer 3)	Cation efflux	Cation transporter; *Acidithiobacillus ferrooxidans*; 49% (77%), 2e-04; WP_067711184	n.d.	n.d.

BXT84_00705	129991-130296	101	Cation transporter of cation diffusion facilitator family transporter	Transport of divalent cations of cobalt, cadmium and/or zinc. These integral membrane proteins were found to increase tolerance to divalent metal ions such as cadmium, zinc, and cobalt. These proteins are thought to be efflux pumps that remove these ions from cells[14]	Cation transporter; *Alicyclobacillus macrosporangiidus*; 45% (93%), 2e-16; WP_029421074	n.d.	n.d.
BXT84_00710	Complement (131122-130526)	198	2-heptaprenyl-1,4-naphthoquinone methyltransferase	S-adenosylmethionine-dependent methyltransferases use S-adenosyl-L-methionine as a substrate for methyltransfer, creating the product S-adenosyl-L homocysteine[15]	SAM-dependent methyltransferase; *S. thermosulfidooxidans*; 47% (96%), 2e-60; WP_020374132	n.d.	n.d.
BXT84_00715	Complement (131186-131533)	115	Hypothetical protein	-	Hypothetical protein A2Y60_06545; *Chloroflexi* bacterium RBG_13_54_9; 49% (51%), 6e-07; OGO03597	n.d.	n.d.
BXT84_00720	131715-132134	139	FAD-dependent pyridine nucleotide-disulphide oxidoreductase	Uses the isoalloxazine ring of FAD to shuttle reducing equivalents from NAD(P)H to a Cys residue that is usually a part of a redox-active disulphide bridge[16]	Pyridine nucleotide-disulphide oxidoreductase; *Lutibacter* sp. BRH_c52; 51% (99%), 7e-44; KUO65327	n.d.	n.d.
BXT84_00725	132750-133103	117	Hypothetical protein	-	Hypothetical protein; *S. acidophilus* TPY; 40% (89%), 2e-10; AEJ40479	n.d.	n.d.
BXT84_00730	133149-134537	462	Integrase	Putative transposase OrfB. Transposase binds to the end of a transposon and catalyzes the movement of the transposon to another part of the genome by a cut and paste mechanism or a	Transposase; *S. acidophilus* TPY; 66% (97%), 0.0; AEJ41300	n.d.	n.d.

				replicative transposition mechanism			
BXT84_00735	134534-135352	272	AAA family ATPase	Members of the AAA+ ATPases function as molecular chaperons, ATPase subunits of proteases, helicases, or nucleic-acid stimulated ATPases	ISChy3, orf3; *S. acidophilus* TPY; 60% (99%), 1e-107; AEJ40482	n.d.	n.d.
BXT84_00740	135379-135861	160	Hypothetical protein	No significant similarity found			
BXT84_00745	136895-137101	68	Hypothetical protein	-	Hypothetical protein; *Bacillus* sp. 1NLA3E; 35% (92%), 2e-06; WP_015595684	n.d.	n.d.
BXT84_00750	137098-138126	342	Hypothetical protein; sensor histidine kinase	A two-component signal transduction system to detect and respond to changes in the environment. Sensor histidine kinase autophosphorylates a histidine residue on detecting an external stimulus. The phosphate is then transferred to an invariant aspartate residue in a highly conserved receiver domain of the response regulator. Phosphorylation activates a variable effector domain of the response regulator, which triggers the cellular response[17]	Sensor histidine kinase; *Moorella mulderi*; 36% (88%), 8e-47; WP_062283913	n.d.	n.d.
BXT84_00755	138093-138818	241	Two-component sensor histidine kinase		DNA-binding response regulator; *Chloroflexi* bacterium 13_1_40CM_68_21; 45% (92%), 1e-55; OLC21636	n.d.	n.d.
BXT84_00760	139080-139862	260	Hypothetical protein	-	Hypothetical protein; *Thermo-plasmatales archaeon* Gpl; 38% (98%), 5e-50; EQB69311	n.d.	n.d.
BXT84_00765	140100-140741	213	Hypothetical protein Methyltrans-ferase type 11	Uses S-adenosyl-L-methionine as a substrate for methyltransfer, creating the product S-adenosyl-L homocysteine[15]	SAM-dependent methyltransferase; *Acidibacillus ferrooxidans*; 46% (91%), 5e-60; WP_067711200	n.d.	n.d.

BXT84_00770	140743-141519	258	Hypothetical protein, rusticyanin	Rusticyanin is a copper-containing protein (cupredoxin, or blue-copper protein) involved in electron-transfer	Cupredoxin; *Acidibacillus ferrooxidans*; 43% (68%), 5e-38; WP_067564969	n.d.	n.d.
BXT84_00775	141847-142089	80	Hypothetical protein	Electron transport	Conserved hypothetical protein (electron transporter rnfE); *Thiolapillus brandeum*; 41% (82%), 3e-07; BAO44383	n.d.	n.d.
BXT84_00780	142584-142766	60	Hypothetical protein with integrase core domain	Binds to the end of a transposon and catalyzes the movement of the transposon to another part of the genome by a cut and paste mechanism or a replicative transposition mechanism. These transposases are found in the planctomycete *Rhodopirellula baltica*, the cyanobacterium Nostoc, and the Gram-positive bacterium *Streptomyces*[13]	Hypothetical protein UY95_C0012G0013, partial; *Parcubacteria* group bacterium GW2011_GWA2_56_7; 56% (95%), 3e-14; KKW44958	n.d.	n.d.
BXT84_00785	143051-143398	115	Hypothetical protein; Rhodonase-like	Proposed to transfer a sulfur atom from thiosulfate to sulfur acceptors like thiol proteins (RSH) with the production of sulfite in *A. caldus*[18]	Rhodanese-like domain-containing protein; *Sulfobacillus thermosulfidooxidans*; 47% (97%), 2e-33; WP_020375927	n.d.	n.d.
BXT84_00790	143661-143864	67	Hypothetical protein	-	Hypothetical protein AKJ57_04905; candidate divison MSBL1 archaeon SCGC-AAA259A05; 35% (91%), 6e-05; KXA89778	n.d.	n.d.
BXT84_00795	143992-144309	105	Hypothetical protein	-	Predicted protein; *Bathycoccus prasinos*; 36% (52%), 0.31; XP_007513050	n.d.	n.d.

BXT84 _00800	Complement (144847-145296)	149	Hypothetical protein	-	Hypothetical protein; *S. thermosulfidooxidans*; 45% (93%), 1e-30; WP_053959794	n.d.	n.d.
BXT84 _00805	145487-146143	218	Hypothetical protein		No significant similarity found		
BXT84 _00810	146211-146417	68	Heavy metal-binding domain-containing protein CopZ	Copper chaperone. Binding and transfer of metal ions, such as copper, cadmium, cobalt and zinc[19]	Heavy metal transport/ detoxification protein; *S. acidophilus* TPY; 70% (97%), 5e-23; AEJ40974	n.d.	n.d.
BXT84 _00815	146459-148927	822	CopA	Lead, cadmium, zinc and mercury transporting ATPase/ copper-translocating P-type ATPase. Pumps copper and other ions out and into cells[20]	Copper-translocating P-type ATPase; *S. thermosulfidooxidans*; 60% (96%), 0.0; WP_020375255	n.d.	n.d.
BXT84 _00820	Complement (149076-149243)	55	Hypothetical protein, CtpA		Copper-translocating P-type ATPase; *Hyphomicrobium* sp. 99; 55% (87%), 3e-09; WP_045835924	n.d.	n.d.
BXT84 _00825	149290-149610	106	Hypothetical protein	-	Hypothetical protein TPY_0271; *S. acidophilus* TPY; 67% (42%), 4e-08; AEJ38473	n.d.	n.d.
BXT84 _00830	149601-149999	132	Hypothetical protein, CsoR	Transcriptional regulators CsoR (copper-sensitive operon repressor) respond to stressors including Cu(I), Ni(I), sulfite, and formaldehyde. Some CsoR also sense other metal ions such as Cu(II), Zn(II), Ag(I), Cd(II) and Ni(II)[21]	Hypothetical protein; *S. acidophilus* TPY; 73 (84%), 3e-53; AEJ41764	n.d.	n.d.
BXT84 _00835	150103-151245	380	Hypothetical protein	-	Hypothetical protein; *Acidibacillus ferrooxidans*; 42% (90%), 8e-77; WP_067711179	n.d.	n.d.

BXT84_00840	151428-151634	68	Hypothetical protein	-	AhpC/TSA family protein; *Alteribacillus bidgolensis*; 33% (57%), 0.005; SDJ12717	n.d.	n.d.
BXT84_00845	152408-153967	519	Hypothetical protein	-	Hypothetical protein; *Brevibacillus choshinensis*; 25% (95%), 1e-36; WP_055748171	n.d.	n.d.
BXT84_00850	153954-155840	628	Tn7-like transposition protein D	TnsABC makes up the core transposition machinery that is incapable of transposition without one of two target site selecting proteins, TnsD or TnsE[22]	Transposase; *Thermotalea metallivorans*; 36% (97%), 4e-130; WP_068557760	n.d.	n.d.
BXT84_00855	155853-157505	550	Tn7-like transposition protein C		Tn7-like transposition protein C; *Geminocystis* sp. NIES-3708; 62% (75%), 0.0; WP_066344860	n.d.	n.d.
BXT84_00860	157526-159688	720	Transposon Tn7 transposition protein tnsB		Hypothetical protein; *Geminocystis* sp. NIES-3708; 49% (99%), 0.0; WP_066344861	n.d.	n.d.
BXT84_00865	159685-160518	277	Tn7-like transposition protein A		Tn7-like transposition protein A; *Geminocystis* sp. NIES-3708; 50% (95%), 1e-87; BAQ60815	n.d.	n.d.

[a]*S. thermotolerans* Kr1 homologs of the plasmid-encoded proteins of *S. thermotolerans* L15 and Y0017 are indicated in red.
[b]Homologs probably involved in metal resistance are indicated in blue.
[c]N.d., no significant amino acid identity with *S. thermotolerans* pL15 and pY0017 ORFs was determined.
[d]References:

1. Deane, S. M. & Rawlings, D. E. Two large, related, cryptic plasmids from geographically distinct isolates of *Sulfobacillus thermotolerans*. *Appl. Environ. Microbiol.* **77,** 8175-8180 (2011).
2. Tie, J. K. & Stafford, D. W. Structure and function of vitamin K epoxide reductase. *Vitam. Horm.* **78,** 103-130 (2008).
3. Paterson, E. S. *et al.* Genetic analysis of the mobilization and leading regions of the IncN plasmids pKM101 and pCU1. *J. Bacteriol.* **181,** 2572-2583 (1999).
4. Hofreuter, D. & Haas, R. Characterization of two cryptic *Helicobacter pylori* plasmids: a putative source for horizontal gene transfer and gene shuffling. *J. Bacteriol.* **184,** 2755-2766 (2002).
5. Garcillán-Barcia, M. P., Francia, M. V. & de la Cruz, F. The diversity of conjugative relaxases and its application in plasmid classification. *FEMS Microbiol. Rev.* **33,** 657-687 (2009).
6. Hamilton, C. M. *et al.* TraG from RP4 and TraG and VirD4 from Ti plasmids confer relaxosome specificity to the conjugal transfer system of pTiC58. *J. Bacteriol.* **182,** 1541-1548 (2000).

7. Morton, T. M., Eaton, D. M., Johnston, J. L. & Archer, G. L. DNA sequence and units of transcription of the conjugative transfer gene complex (trs) of *Staphylococcus aureus* plasmid pGO1. *J. Bacteriol.* **175,** 4436-4447 (1993).
8. Bork, P., Sander, C. & Valencia, A. An ATPase domain common to prokaryotic cell cycle proteins, sugar kinases, actin, and hsp70 heat shock proteins. *Proc. Natl. Acad. Sci. USA* **89,** 7290-7294 (1992).
9. Wallden, K., Rivera-Calzada, A. & Waksman, G. Type IV secretion systems: versatility and diversity in function. *Cell Microbiol.* **12,** 1203-1212 (2010).
10. Scheurwater, E., Reid, C. W. & Clarke, A. J. Lytic transglycosylases: bacterial space-making autolysins. *Int. J. Biochem. Cell Biol.* **40,** 586-591 (2008).
11. Aravind, L., Leipe, D. D. & Koonin, E. V. Toprim--a conserved catalytic domain in type IA and II topoisomerases, DnaG-type primases, OLD family nucleases and RecR proteins. *Nucleic Acids Res.* **26,** 4205-4213 (1998).
12. Kikuchi, S., Shibuya, I. & Matsumoto, K. Viability of an *Escherichia coli* pgsA null mutant lacking detectable phosphatidylglycerol and cardiolipin. *J. Bacteriol.* **182,** 371-376 (2000).
13. DasSarma, S. Identification and analysis of the gas vesicle gene cluster on an unstable plasmid of *Halobacterium halobium. Experientia* **49,** 482-486 (1993).
14. Xiong, A. & Jayaswal, R. K. Molecular characterization of a chromosomal determinant conferring resistance to zinc and cobalt ions in *Staphylococcus aureus*. *J. Bacteriol.* **180,** 4024-4029 (1998).
15. Wooderchak, W. L., Zhou, Z. S. & Hevel, J. Assays for S-adenosylmethionine (AdoMet/SAM)-dependent methyltransferases. *Curr. Protoc. Toxicol.* **4,** Unit4.26 (2008).
16. Kuriyan, J. *et al.* Convergent evolution of similar function in two structurally divergent enzymes. *Nature* **352,** 172-174 (1991).
17. Kenney, L. J. How important is the phosphatase activity of sensor kinases? *Curr. Opin. Microbiol.* **13,** 168-176 (2010).
18. Guo, X. *et al.* Comparative genome analysis reveals metabolic versatility and environmental adaptations of S*ulfobacillus thermosulfidooxidans* strain ST. *PLoS One* **9,** e99417; 10.1371/journal.pone.0099417 (2014).
19. Jordan, I. K., Natale, D. A. & Galperin, M. Y. Copper chaperones in bacteria: association with copper-transporting ATPases. *Trends Biochem. Sci.* **25,** 480-481 (2000).
20. Rensing, C., Fan, B., Sharma, R., Mitra, B. & Rosen, B. P. CopA: An *Escherichia coli* Cu(I)-translocating P-type ATPase. *Proc. Natl. Acad. Sci. U S A* **97,** 652-656 (2000).
21. Liu, T. *et al.* CsoR is a novel *Mycobacterium tuberculosis* copper-sensing transcriptional regulator. *Nat. Chem. Biol.* **3,** 60-68 (2007).
22. Peters, J. E. & Craig, N. L. Tn7: smarter than we thought. *Nat. Rev. Mol. Cell Biol.* **2,** 806-814 (2001).

Table S3. Enzymes of the TCA cycle and glyoxylate cycle encoded in the genome of *Sulfobacillus thermotolerans* Kr1

CDS (Protein ID)	KO	Protein size, a. a.	Putative homologs	Score
BXT84_11310	K01647	375	CS; citrate synthase [EC:2.3.3.1]	353
BXT84_12440	K01681	900	ACO; aconitate hydratase [EC:4.2.1.3]	789
BXT84_09895	K00031	405	IDH1; isocitrate dehydrogenase [EC:1.1.1.42]	410
BXT84_08405	K00164	932	OGDH; 2-oxoglutarate dehydrogenase E1 component [EC:1.2.4.2]	658
BXT84_08400	K00658	414	DLST; 2-oxoglutarate dehydrogenase E2 component (dihydrolipoamide succinyltransferase) [EC:2.3.1.61]	312
BXT84_01035<br>BXT84_00975<br>BXT84_01885<br>BXT84_13485	K00382	474<br>468<br>480<br>466	DLD; dihydrolipoamide dehydrogenase [EC:1.8.1.4]	179<br>333<br>143<br>202
BXT84_14445	K00174	581	korA; 2-oxoglutarate/2-oxoacid ferredoxin oxidoreductase subunit alpha [EC:1.2.7.3 1.2.7.11]	382
BXT84_14450	K00175	287	korB; 2-oxoglutarate/2-oxoacid ferredoxin oxidoreductase subunit beta [EC:1.2.7.3 1.2.7.11]	248
BXT84_09910	K01902	289	sucD; succinyl-CoA synthetase alpha subunit [EC:6.2.1.5]	254
BXT84_09905	K01903	370	sucC; succinyl-CoA synthetase beta subunit [EC:6.2.1.5]	275
BXT84_05565	K00239	591	sdhA; succinate dehydrogenase / fumarate reductase, flavoprotein subunit [EC:1.3.5.1 1.3.5.4]	417
BXT84_05570	K00240	250	sdhB; succinate dehydrogenase / fumarate reductase, iron-sulfur subunit [EC:1.3.5.1 1.3.5.4]	209
BXT84_12860	K01679	465	E4.2.1.2B; fumarate hydratase, class II [EC:4.2.1.2]	320
BXT84_09900	K00024	309	mdh; malate dehydrogenase [EC:1.1.1.37]	273
BXT84_05220	K01638	526	aceB; malate synthase [EC:2.3.3.9]	436

*KO, KEGG Orthology identifiers. Ortholog annotation was carried out using KOALA (KEGG Orthology and Links Annotation) system. Genome annotation was performed using Prokka v1.10 and the NCBI Prokaryotic Genome Annotation Pipeline (United States, http://www.ncbi.nlm.nih.gov/genome/annotation_prok/).

Table S4. Enzymes of the methylcitrate cycle encoded in the genome of *Sulfobacillus thermotolerans* Kr1

CDS (Protein ID)	KO*	Protein size, a. a.	Putative homologs	Score
BXT84_06180	K03417	304	**prpB; methylisocitrate lyase [EC:4.1.3.30]****	268
BXT84_05570	K00240	250	sdhB; succinate dehydrogenase / fumarate reductase, iron-sulfur subunit [EC:1.3.5.1 1.3.5.4]	209
BXT84_05565	K00239	591	sdhA; succinate dehydrogenase / fumarate reductase, flavoprotein subunit [EC:1.3.5.1 1.3.5.4]	417
BXT84_12860	K01679	465	E4.2.1.2B; fumarate hydratase, class II [EC:4.2.1.2]	320
BXT84_09900	K00024	309	mdh; malate dehydrogenase [EC:1.1.1.37]	273
BXT84_06190	K01647	370	**prpC; 2-methylcitrate synthase (EC 2.3.3.5)**	371
BXT84_06185	K01720	478	**prpD; 2-methylcitrate dehydratase [EC:4.2.1.79]**	584
BXT84_12440	K01681	900	ACO; aconitate hydratase [EC:4.2.1.3]	789

*KO, KEGG Orthology identifiers. Ortholog annotation was carried out using KOALA (KEGG Orthology and Links Annotation) system. Genome annotation was performed using Prokka v1.10 and the NCBI Prokaryotic Genome Annotation Pipeline (United States, http://www.ncbi.nlm.nih.gov/genome/annotation_prok/).
**The key enzymes of the methylcitrate cycle are indicated in bold.

Table S5. Proteins of the oxalate degradation pathway encoded in the genome of *Sulfobacillus thermotolerans* Kr1

CDS (Protein ID)	KO*	Protein size, a. a.	Putative homologs	Score
BXT84_15730	K01577	559	oxc; oxalyl-CoA decarboxylase [EC:4.1.1.8]	490
BXT84_15735	K07749	416	frc; formyl-CoA transferase [EC:2.8.3.16]	632
BXT84_15755	K08177	463	oxlT; MFS transporter, OFA family, oxalate/formate antiporter	39

*KO, KEGG Orthology identifiers. Ortholog annotation was carried out using KOALA (KEGG Orthology and Links Annotation) system. Genome annotation was performed using Prokka v1.10 and the NCBI Prokaryotic Genome Annotation Pipeline (United States, http://www.ncbi.nlm.nih.gov/genome/annotation_prok/).

Table S6. Electron transfer components encoded in the genome of *Sulfobacillus thermotolerans* Kr1

CDS (Protein ID)	KO*	Protein size, a. a.	Putative homologs	Score
BXT84_08145	K06196	237	ccdA; cytochrome c-type biogenesis protein	110
BXT84_05350	K02198	680	ccmF; cytochrome c-type biogenesis protein CcmF	400
BXT84_05345	K02197	135	ccmE; cytochrome c-type biogenesis protein CcmE	53
BXT84_09420	-	183	Sulfocyanin (SoxE)	8
BXT84_08470	-	176	Sulfocyanin	9
BXT84_09420	-	183	Sulfocyanin	8
BXT84_00770	-	258	Rusticyanin	8
BXT84_05090	-	161	Rusticyanin	10
BXT84_06075	-	209	Cytochrome_b6	-
BXT84_07665	K00412	212	Cytochrome_b6; ubiquinol-cytochrome c reductase cytochrome b subunit	11
BXT84_03390	-	205	Cytochrome_b6	-
BXT84_03905	K00425	465	cydA; cytochrome d ubiquinol oxidase subunit I [EC:1.10.3.14]	450
BXT84_03900	K00426	333	cydB; cytochrome d ubiquinol oxidase subunit II [EC:1.10.3.14]	297
BXT84_04585	K00426	335	cydB; cytochrome d ubiquinol oxidase subunit II [EC:1.10.3.14]	324
BXT84_04580	K00425	455	cydA; cytochrome d ubiquinol oxidase subunit I [EC:1.10.3.14]	351
BXT84_04115	K00426	336	cydB; cytochrome d ubiquinol oxidase subunit II [EC:1.10.3.14]	211
BXT84_04110	K00425	471	cydA; cytochrome d ubiquinol oxidase subunit I [EC:1.10.3.14]	422
BXT84_08270	K00343	473	nuoN; NADH-quinone oxidoreductase subunit N [EC:1.6.5.3]	251
BXT84_08265	K00342	480	nuoM; NADH-quinone oxidoreductase subunit M [EC:1.6.5.3]	231
BXT84_08260	K00341	621	nuoL; NADH-quinone oxidoreductase subunit L [EC:1.6.5.3]	350
BXT84_03500	K00341	551	nuoL; NADH-quinone oxidoreductase subunit	87

			L [EC:1.6.5.3]	
BXT84_08255	K00340	100	nuoK; NADH-quinone oxidoreductase subunit K [EC:1.6.5.3]	64
BXT84_03505	K00340	99	nuoK; NADH-quinone oxidoreductase subunit K [EC:1.6.5.3]	27
BXT84_08250	K00339	195	nuoJ; NADH-quinone oxidoreductase subunit J [EC:1.6.5.3]	38
BXT84_03510	K00339	159	nuoJ; NADH-quinone oxidoreductase subunit J [EC:1.6.5.3]	26
BXT84_03515	K00338	135	nuoI; NADH-quinone oxidoreductase subunit I [EC:1.6.5.3]	29
BXT84_08245	K00338	151	nuoI; NADH-quinone oxidoreductase subunit I [EC:1.6.5.3]	75
BXT84_08240	K00337	329	nuoH; NADH-quinone oxidoreductase subunit H [EC:1.6.5.3]	315
BXT84_03520	K00337	300	nuoH; NADH-quinone oxidoreductase subunit H [EC:1.6.5.3]	180
BXT84_08235	K00336	772	nuoG; NADH-quinone oxidoreductase subunit G [EC:1.6.5.3]	212
BXT84_15710	K00335	403	nuoF; NADH-quinone oxidoreductase subunit F [EC:1.6.5.3]	177
BXT84_08225	K00334	160	nuoE; NADH-quinone oxidoreductase subunit E [EC:1.6.5.3]	72
BXT84_08220	K00333	402	nuoD; NADH-quinone oxidoreductase subunit D [EC:1.6.5.3]	454
BXT84_03525	K00333	371	nuoD; NADH-quinone oxidoreductase subunit D [EC:1.6.5.3]	284
BXT84_08215	K00332	153	nuoC; NADH-quinone oxidoreductase subunit C [EC:1.6.5.3]	109
BXT84_03530	K00332	124	nuoC; NADH-quinone oxidoreductase subunit C [EC:1.6.5.3]	19
BXT84_08210	K00331	171	nuoB; NADH-quinone oxidoreductase subunit B [EC:1.6.5.3]	174
BXT84_03535	K00331	181	nuoB; NADH-quinone oxidoreductase subunit B [EC:1.6.5.3]	178
BXT84_08205	K00330	117	nuoA; NADH-quinone oxidoreductase subunit A [EC:1.6.5.3]	79
BXT84_02290	K02276	181	coxC; cytochrome c	14

			oxidase subunit III [EC:1.9.3.1]	
BXT84_02285	K02274	623	coxA; cytochrome c oxidase subunit I [EC:1.9.3.1]	323
BXT84_02280	K02275	272	coxB; cytochrome c oxidase subunit II [EC:1.9.3.1]	54
BXT84_15375	K02276	189	coxC; cytochrome c oxidase subunit III [EC:1.9.3.1]	17
BXT84_15380	K02274	649	coxA; cytochrome c oxidase subunit I [EC:1.9.3.1]	289
BXT84_15385	K02275	270	coxB; cytochrome c oxidase subunit II [EC:1.9.3.1]	59
BXT84_07725	K02276	189	coxC; cytochrome c oxidase subunit III [EC:1.9.3.1]	20
BXT84_07720	K02274	638	coxA; cytochrome c oxidase subunit I [EC:1.9.3.1]	307
BXT84_07715	K02275	273	coxB; cytochrome c oxidase subunit II [EC:1.9.3.1]	56
BXT84_04615	K02275	270	coxB; cytochrome c oxidase subunit II [EC:1.9.3.1]	59
BXT84_04620	K02274	649	coxA; cytochrome c oxidase subunit I [EC:1.9.3.1]	289
BXT84_04625	K02276	189	coxC; cytochrome c oxidase subunit III [EC:1.9.3.1]	17
BXT84_13935	K02300	90	cyoD; cytochrome o ubiquinol oxidase subunit IV, AA3-600 quinol oxidase subunit IV	14
BXT84_13930	K02828	206	qoxC; cytochrome aa3-600 menaquinol oxidase subunit III [EC:1.10.3.12]	194
BXT84_13925	K02827	655	qoxB; cytochrome aa3-600 menaquinol oxidase subunit I [EC:1.10.3.12]	747
BXT84_13920	-	294	cytochrome ubiquinol oxidase subunit II	-

*KO, KEGG Orthology identifiers. Ortholog annotation was carried out using KOALA (KEGG Orthology and Links Annotation) system. Genome annotation was performed using Prokka v1.10 and the NCBI Prokaryotic Genome Annotation Pipeline (United States, http://www.ncbi.nlm.nih.gov/genome/annotation_prok/).

Table S7. Proteins predicted to participate in stress resistance and defense systems of *Sulfobacillus thermotolerans* Kr1

CDS (Protein ID)	KO*	Protein size, a. a.	Putative homologs	Score
Oxidative stress defense proteins				
BXT84_04395	K04564	199	SOD2; superoxide dismutase, Fe-Mn family [EC:1.15.1.1]	153
BXT84_05155	K00432	157	gpx; glutathione peroxidase [EC:1.11.1.9]	127
BXT84_12315	K03386	215	PRDX2_4; peroxiredoxin (alkyl hydroperoxide reductase subunit C) [EC:1.11.1.15]	186
BXT84_15165	K03564	153	BCP; peroxiredoxin Q/BCP [EC:1.11.1.15]	120
BXT84_04275	K11065	171	tpx; thiol peroxidase, atypical 2-Cys peroxiredoxin [EC:1.11.1.15]	139
BXT84_15685	-	178	thioredoxin peroxidase	140
Metal resistance system				
BXT84_08345	K06147	628	ABCB-BAC; ATP-binding cassette, subfamily B, bacterial	525
BXT84_08350	K06147	575	ABCB-BAC; ATP-binding cassette, subfamily B, bacterial	476
BXT84_13640	K18889	585	mdlA; ATP-binding cassette, subfamily B, multidrug efflux pump	472
BXT84_13645	K18890	594	mdlB; ATP-binding cassette, subfamily B, multidrug efflux pump	450
BXT84_00115	K06147	613	ABCB-BAC; ATP-binding cassette, subfamily B, bacterial	149
BXT84_01235	K06158	530	ABCF3; ATP-binding cassette, subfamily F, member 3	162
BXT84_04120	K16013	589	cydD; ATP-binding cassette, subfamily C, bacterial CydD	314
BXT84_04125	K16012	572	cydC; ATP-binding cassette, subfamily C, bacterial CydC	265
BXT84_05225	K18890	610	mdlB; ATP-binding cassette, subfamily B, multidrug efflux pump	307
BXT84_05230	K18889	580	mdlA; ATP-binding cassette, subfamily B, multidrug efflux pump	289
BXT84_01960	K08221	393	yitG; MFS transporter, ACDE family, multidrug resistance protein	248
BXT84_02625	K08369	450	ydjE; MFS transporter, putative metabolite:H+ symporter	176
BXT84_02945	K08369	432	ydjE; MFS transporter, putative metabolite:H+ symporter	293
BXT84_04195	K08368	425	yaaU; MFS transporter, putative metabolite transport protein	117
BXT84_07055	K08368	459	yaaU; MFS transporter, putative metabolite transport protein	42
BXT84_08045	K08222	392	yqgE; MFS transporter, YQGE family, putative transporter	54
BXT84_09605	K08368	446	yaaU; MFS transporter, putative metabolite transport protein	151
BXT84_11095	K08369	454	ydjE; MFS transporter, putative metabolite:H+ symporter	230
BXT84_11620	K08221	448	yitG; MFS transporter, ACDE family, multidrug resistance protein	50
BXT84_13445	K08222	399	yqgE; MFS transporter, YQGE family, putative transporter	75
BXT84_00815	K17686	822	copA; Cu+-exporting ATPase [EC:3.6.3.54]	421
BXT84_04925	K17686	814	copA; Cu+-exporting ATPase [EC:3.6.3.54]	266

BXT84_07130	K17686	817	copA; Cu+-exporting ATPase [EC:3.6.3.54]	405
BXT84_07420	K01551	184	arsA; arsenite-transporting ATPase [EC:3.6.3.16]	28
BXT84_00810	K07213	68	ATOX1; copper chaperone	17
BXT84_13290	K16264	302	czcD; cobalt-zinc-cadmium efflux system protein	116
BXT84_14580	K02032	333	ABC.PE.A1; peptide/nickel transport system ATP-binding protein	154
BXT84_14585	K02031	328	ABC.PE.A; peptide/nickel transport system ATP-binding protein	102
BXT84_14590	K02034	304	ABC.PE.P1; peptide/nickel transport system permease protein	156
BXT84_14595	K02033	338	ABC.PE.P; peptide/nickel transport system permease protein	223
BXT84_14600	K02035	601	ABC.PE.S; peptide/nickel transport system substrate-binding protein	153
BXT84_13510	-	252	Polyphosphate kinase	203
BXT84_05995	K01524	521	ppx-gppA; exopolyphosphatase / guanosine-5'-triphosphate,3'-diphosphate pyrophosphatase [EC:3.6.1.11 3.6.1.40]	135
BXT84_02885	K07658	236	phoB1; two-component system, OmpR family, alkaline phosphatase synthesis response regulator PhoP	136
BXT84_02890	K07636	460	phoR; two-component system, OmpR family, phosphate regulon sensor histidine kinase PhoR [EC:2.7.13.3]	164
BXT84_02895	K02040	275	pstS; phosphate transport system substrate-binding protein	62
BXT84_02900	K02037	291	pstC; phosphate transport system permease protein	103
BXT84_02905	K02038	283	pstA; phosphate transport system permease protein	115
BXT84_02910	K02036	264	pstB; phosphate transport system ATP-binding protein [EC:3.6.3.27]	202
BXT84_02915	K02039	218	phoU; phosphate transport system protein	170
BXT84_03480	K00520	543	merA; mercuric reductase [EC:1.16.1.1]	433
BXT84_07790	K03892	105	arsR; ArsR family transcriptional regulator	56
BXT84_07420	K01551	184	arsA; arsenite-transporting ATPase [EC:3.6.3.16]	28
BXT84_07430	K03893	430	arsB; arsenical pump membrane protein	335

*KO, KEGG Orthology identifiers. Ortholog annotation was carried out using KOALA (KEGG Orthology and Links Annotation) system. Genome annotation was performed using Prokka v1.10 and the NCBI Prokaryotic Genome Annotation Pipeline (United States, http://www.ncbi.nlm.nih.gov/genome/annotation_prok/).
